# CDK9 degrader induces BRCAness and sensitizes castration-resistant prostate cancer to PARP inhibitor

**DOI:** 10.7150/thno.131907

**Published:** 2026-06-17

**Authors:** Jiaxuan Li, Jingya Sun, Weisong Tan, Guoqi Li, Wenjie Xiao, Jiajun Wu, Wei Yang, Changqing Chen, Yang Li, Jiakuan Liu, Yuanyu Liu, Dong Liu, Xiao-hua Chen, Rujian Zhu, Ruimin Huang, Jun Yan

**Affiliations:** 1 Department of Urology, Shanghai Pudong Hospital, Fudan University Pudong Medical Center; Laboratory Animal Center, Fudan University, Shanghai 200032, China.; 2 Shanghai Institute of Infectious Disease and Biosecurity, School of Public Health, Fudan University, Shanghai 200032, China.; 3 Shanghai Institute of Materia Medica, Chinese Academy of Sciences, Shanghai 201203, China.; 4 Laboratory Animal Center, Fudan University, Shanghai 200032, China.; 5 School of Chinese Materia Medica, Nanjing University of Chinese Medicine, Nanjing 210023, China.; 6 University of Chinese Academy of Sciences, Beijing 100049, China.; 7 State Key Laboratory of Genetic Engineering, School of Life Sciences, Fudan University, Shanghai 200438, China.; 8 School of Pharmaceutical Science and Technology, Hangzhou Institute for Advanced Study, University of Chinese Academy of Sciences, Hangzhou 310024, China.

**Keywords:** castration-resistant prostate cancer, BRCAness, CDK9 protein degrader, PARP inhibitor, homologous recombination repair

## Abstract

**Rationale:**

Castration-resistant prostate cancer (CRPC) poses significant therapeutic challenges due to its aggressive nature and limited effective treatments. Although PARP inhibitor olaparib has been approved for metastatic CRPC patients bearing BRCA1/2 mutations, its application is confined to this specific patient subpopulation. The induction of “BRCAness” feature in CRPC patients without BRCA1/2 mutations becomes a significant challenge.

**Methods:**

A transcriptomic analysis to identify potential “BRCAness” regulator was performed on 921 prostate cancer (PCa) patients from 6 public datasets and validated in our own cohort. D45, a selective small-molecule protein degrader for cyclin-dependent kinase 9 (CDK9), alone or combined with olaparib, was applied in CRPC cell lines (C4-2 and 22Rv1) lacking BRCA1/2 mutations for cell viability, colony formation and apoptosis-related assays. “BRCAness” phenotype was characterized by Western blotting and γH2AX foci accumulation assays. RNA-seq and CUT&Tag assays were used to reveal how D45 regulated homologous recombination repair (HRR)-related genes.

**Results:**

We found the transcriptional regulator CDK9 was overexpressed in CRPC and correlated with advanced Gleason scores, metastasis, and poor prognosis. D45 treatment decreased cell survival, and led to downregulation of HRR-related genes (BRCA1/2 and RAD51) with the reduced recruitment of phosphorylated-RNA polymerase II (pSer2) to the ends of these genes with increased DNA damage, indicating a “BRCAness” phenotype induction. The synthetic vulnerability synergized with D45 plus olaparib was thus tested *in vitro*, showing the enhanced apoptosis in CRPC cells. Moreover, sequential D45 and olaparib administration significantly suppressed 22Rv1 xenograft growth* in vivo* (*P* < 0.001), reduced RAD51 expression and increased DNA damage. Toxicity was tolerable and consistent with prior reports. Their synergistic effect was confirmed in *ex vivo* explants from human PCa specimens.

**Conclusions:**

Our findings demonstrated CDK9 as a master regulator of BRCAness and proposed targeting CDK9 as a potential strategy to sensitize CRPC patients without BRCA1/2 mutations to PARP inhibition.

## Introduction

Prostate cancer (PCa) remains the most prevalent malignancy and one of the leading causes of cancer-related death in men worldwide, with castration-resistant prostate cancer (CRPC) representing the lethal form driven by persistent androgen receptor (AR) signaling and adaptive survival mechanisms 1-3. About 84% of CRPC patients advance to metastatic stage, while conventional treatments for metastatic CRPC (mCRPC) patients often prove inadequate, resulting in a 5-year survival rate of only ~30% 4. In light of these challenges, development of novel and more effective therapeutic strategies for CRPC is urgent.

Next-generation sequencing reveals that ~25% of mCRPC patients harbor homologous recombination repair (HRR) gene defects, including *BRCA2* (8.7%), *ATM* (5.9%), and *BRCA1* (1-6.3%) mutations, conferring “BRCAness” vulnerability to poly (ADP-ribose) polymerase (PARP) inhibitor (PARPi) via synthetic lethality 5-9, which occurs between two specific genes when the perturbation of either gene alone is viable but the perturbation of both genes simultaneously leads to cell death 10, 11. PARPi could prevent DNA repair, resulting in the accumulation of DNA damage. Olaparib, as a well-known PARPi, has already been approved for HRR or *BRCA1/2* gene-mutated mCRPC by FDA, EMA and NMPA in the period 2020-2021 12, 13; and rucaparib, another PARPi, was also approved by FDA for BRCA-mutated mCRPC in 2020 14. Even though PARPis have improved progression-free survival in biomarker-selected cohorts, their clinical utility remains constrained: only 12-20% of CRPC patients exhibit *BRCA1/2* alterations, and acquired resistance via HRR restoration frequently limits therapeutic durability 8. These results reveal the following limitations of PARPi application on CRPC in clinic: 1) it is only applicable in patient harboring specific genetic defects; 2) single-agent of PARPi has limited efficacy; 3) acquired resistance has occurred inevitably. To overcome these barriers, strategies inducing homologous recombination deficiency have emerged to sensitize BRCA1/2-proficient tumors 15, 16. Targeting molecular drivers that regulate both CRPC progression and HRR competency could expand PARPi efficacy to broader populations. This approach aims to leverage pharmacological induction of BRCAness, bypassing reliance on pre-existing genetic defects while mitigating resistance mechanisms, thereby redefining precision oncology paradigms for mCRPC.

Cyclin-dependent kinases (CDKs), a family of serine/threonine kinases, orchestrate tumor progression through cell cycle regulation, transcriptional control, and metabolic reprogramming. CDK9, a pivotal transcription-related kinase, drives RNA polymerase II (RNA Pol II)-mediated oncogene expression by phosphorylating the C-terminal domain, a rate-limiting step in transcriptional elongation 17, 18. Elevated expression of CDK9 was found in different types of cancer, such as leukemia, breast cancer and PCa. CDK9 promotes cancer progression mainly through controlling the transcription of *MYC* and *MCL1*, two important genes for cancer cell apoptosis and survival 19, 20. Thus, CDK9 has become a promising target for anticancer therapy, and strategies for blocking CDK9 transcriptional activity have been developed. For example, small-molecule inhibitors of CDK9 were designed to occupy its ATP-binding pocket 21, 22. Pharmacological CDK9 inhibition exhibited broad anticancer potential against renal carcinoma and PCa cells 23, 24. However, some CDK9 inhibitors, such as flavopiridol, suffered from dose-limiting toxicities due to off-target CDK inhibition 25. Benefited from proteolysis targeting chimeras (PROTACs) technique, the development of CDKs protein degraders indicates a promising therapeutic strategy for treating transcription-addicted cancers 26. For example, a PROTAC BSJ-5-63 (targeting CDK12, CDK7 and CDK9) could diminish BRCA1/BRCA2 expression and attenuate AR signaling, sensitizing CRPC to PARP inhibitors and enhancing the therapeutic efficacy 27. Whether these effects are dependent on CDK9 and how CDK9 is involved in “BRCAness” induction, need to be thoroughly investigated in mCRPC without HRR gene defects.

We previously developed a potent and selective CDK9 degrader (D45), which could induce degradation of CDK9 protein, and lead to apoptosis *in vitro* along with xenograft growth inhibition in triple-negative breast cancer 28. In this study, to address the significant challenge in CRPC patients without *BRCA1/2* mutations, *i.e.*, how to induce “BRCAness” state to sensitize patients to PARPi therapy, D45 treatment was tested, showing the “BRCAness” with the downregulation of HRR-genes, including *BRCA1/2*, *PALB2*, and *RAD51*. A sequential regimen of D45 and olaparib was applied in CRPC xenografts *in vivo* and human PCa explants *ex vivo*, and demonstrated the pre-existing DNA damage caused by CDK9 degrader was exacerbated by PARP inhibition, resulting in the accumulation of unrepaired DNA double-strand breaks and ultimate cell death.

## Materials and Methods

### Public data analysis

TCGA-PRAD dataset was obtained from The Cancer Genome Atlas Program (TCGA) database, with clinical information retrieved from cBioPortal (https://www.cbioportal.org). DKFZ-PRAD dataset was sourced from the Prostate Cancer dataset published in Cancer Cell 2018 29 on cBioPortal; human PCa-related datasets (GSE35988, GSE70768, GSE8511, and GSE74367) and CDK9 inhibitor or degrader treatment-related datasets (GSE205363, GSE244716, GSE169090, and GSE282602) were downloaded from the Gene Expression Omnibus (GEO) repository (http://www.ncbi.nlm.nih.gov/geo). For analysis on mRNA expression level in TCGA-PRAD dataset, log_2_(FPKM+1) value of each probe was applied, and average value was used for the genes with multiple probes. The human PCa proteomic datasets OEP004998 and PXD049446 were obtained from the National Omics Data Encyclopedia (https://www.biosino.org/node) and ProteomeCentral (https://proteomecentral.proteomexchange.org/), respectively.

### PCa cell lines and human prostate specimens

Human PCa cell lines 22Rv1 and C4-2 cells were purchased from American Type Culture Collection. Cells were maintained in RPMI1640 medium, supplemented with 10% fetal bovine serum (FBS; ThermoFisher Scientific, USA) and penicillin (100 U/mL) plus streptomycin (100 μg/mL) at 37 °C in 5% CO_2_. Human benign prostatic hyperplasia (BPH), PCa and CRPC specimens were obtained from Shanghai Pudong Hospital. All procedures were performed in compliance with relevant laws and institutional guidelines and have been approved by Ethics Committees of Shanghai Pudong Hospital, Fudan University Pudong Medical Center (2025-KY-019-E01 and 2026-IIT-012-E01). The informed consent was obtained for experimentation with human subjects.

### Small interfering RNA (siRNA) transfection

siRNAs targeting human CDKs and non-specific control siRNA were purchased from Sangon Biotech (China). Following the manufacturer’s protocol, C4-2 cells were plated in 6-well plates at 2 × 10^6^ cells per well or in 96-well plates at 3 × 10^3^ cells per well, and transfected with 40 nM of siRNA duplex using Lipofectamine RNAiMAX (13778150, ThermoFisher Scientific) in Opti-MEM (31985070, ThermoFisher Scientific). 48 h after transfection, cells were used for further experiments. The siRNA sequences were listed in Table S1.

### Animal studies

All animal experiments were approved by the Institutional Animal Care and Use Committee (IACUC) of the Shanghai Institute of Materia Medica, Chinese Academy of Sciences (2020-09-HRM-40 and 2024-06-HRM-79). For spontaneous CRPC model, both *Pb*C*re;Pten^F/F^* and control *Pten^F/F^* mice were maintained on C57BL/6 genetic background and underwent orchiectomy at 4-5 months of age. After 12 months, these mice were euthanized, and the prostate tissues were harvested for histological analysis.

For the fragment implantation of CRPC xenograft model, 1 × 10^7^ 22Rv1 cells were injected subcutaneously on the flanks of 6 to 8-week-old male BALB/c nude mice (GemPharmatech, China). The original subcutaneous tumors were harvested upon reaching a volume of 500 mm^3^, and chopped into small blocks (~ 2 mm^3^) for further subcutaneous implantation via trocar into 6-week-old male BALB/c nude mice. Once the xenograft volume reached 70 mm^3^, 1.5 mg/kg of D45 and/or 50 mg/kg of olaparib (HY-10162, MedChemExpress, China) were intraperitoneally (*ip*) administrated according to the indicated schedule. For the combination treatment group, D45 was injected first, and olaparib was injected 6 h later; single drug or vehicle (PBS) were used as the control groups. The body weight of the mice was recorded and the xenograft volume was measured with a caliper daily. Tumor volumes were calculated using the formula: Volume = (length × width^2^) × 0.5. Two weeks later, the animals were euthanized and the xenografts were harvested for further analyses.

For drug toxicity assessment, 8-week-old male C57BL/6 mice received 1.5 mg/kg of D45 and/or 50 mg/kg of olaparib via* ip* administration according to the indicated schedule for 28 consecutive days. For the combination treatment group, D45 was administered first, followed by olaparib after a 6 h-interval; and control groups received either the single agent or vehicle. Body weight and clinical signs were recorded daily. At the end of the 28-day treatment period, mice were sacrificed, and peripheral blood was collected for complete blood count (CBC) using a hematology analyzer (XN-1000V, Sysmex, Japan) and for serum biochemical analysis using a biochemical analyzer (Cobas C501, Roche, Germany) with the corresponding kits for alanine aminotransferase (ALT), aspartate aminotransferase (AST), creatinine (CREA), and urea. Major organs (heart, liver, lung, kidney, small intestine, and spleen) were harvested for histopathological assessment via hematoxylin and eosin (H&E) staining.

### Immunohistochemistry (IHC) staining

Tissues were fixed in 4% paraformaldehyde, embedded in paraffin, and sectioned at a thickness of 5 μm. The slides were deparaffinized in xylene, rehydrated through graded alcohol and underwent antigen retrieval in citrate buffer (pH 6.0) for 30 min. Endogenous peroxidase activity was blocked with a peroxidase reagent for 10 min, and non-specific binding was blocked with normal goat serum for 10 min (KIT-9707, MXB Biotechnologies, China). Sections were incubated with primary antibodies at 4 °C overnight, followed by the corresponding secondary antibodies at room temperature for 30 min. 3,3'-diaminobenzidine (DAB; MXB Biotechnologies) was used for visualization with hematoxylin counterstaining. The antibody information was listed in Table S2.

### Western blotting assay

Total protein lysates were obtained using RIPA lysis buffer containing protease and phosphatase inhibitors (M5293 and M7528, Abmole, China). 20 µg protein lysates were separated by SDS-PAGE and transferred to 0.45 μm nitrocellulose membranes (Merck Millipore, USA), followed by blocking with TBST containing either 5% skimmed milk powder or 5% bovine serum albumin (A600332, Sangon Biotech) at room temperature for 1 h. After the incubation with primary antibodies at 4 °C overnight, the secondary antibody was incubated at room temperature for 1 h. The signal was detected using the enhanced ECL chemiluminescent substrate (Shanghai Tanon Life Science, China), and the images were captured using the ChemiDoc MP Imaging System (Bio-Rad, USA).

### Reverse transcription-quantitative polymerase chain reaction (RT-qPCR)

Total RNA was extracted by Trizol (15596018, ThermoFisher Scientific) and cDNA was synthesized using Hifair AdvanceFast 1^st^ Strand cDNA Synthesis Kit (11149ES60, YEASEN, China). qPCR was further performed using ChamQ Universal SYBR qPCR Master Mix (Q311-02, Vazyme, China). mRNA expression levels were normalized to *ACTB*, using the 2^-ΔΔCt^ method to calculate the relative expression levels. The primer sequences were listed in Table S1.

### Cell viability assay

22Rv1 and C4-2 cells were seeded in 96-well plates at a density of 3,000 cells/well. Cells were treated with D45 and/or olaparib for 72 h. MTT solution (5 mg/mL) was added and incubated at 37 °C for 4 h. Formazan crystals were dissolved with 100 μL DMSO per well, followed by an incubation at 37 °C for 15 min. Absorbance was measured at 490 nm using a microplate reader, with 680 nm as the reference. The combination index (CI) of D45 and olaparib was calculated using Compusyn software (ComboSyn, UK). The combined drug effects were quantified according to the CI theorem from Chou-Talalay’s method, which defines: additive effect (CI = 1), synergism (CI < 1), and antagonism (CI > 1).

### Colony formation assay

Cells were seeded into 12-well plates at a density of 1,500 cells/well. After 2-3 days, cells were treated with various concentrations of D45 and/or olaparib for 7 days. Colonies were subsequently fixed with 4% formaldehyde, washed with PBS and stained with 0.5% crystal violet solution (Sigma-Aldrich, USA).

### Apoptosis analysis

Cells were seeded in 6-well plates at 1.5 × 10^5^/well, and D45 and/or olaparib at the indicated concentrations were added on the next day for 72 h. Both floating and adherent cells in each well were collected, washed with PBS and stained with FITC-Annexin V in a buffer containing propidium iodide (PI) using FITC Annexin V Apoptosis Detection Kit I (556547, BD Biosciences, USA), followed by flow cytometry (NovoCyte, Agilent Technologies, USA). The Annexin V-positive cells were defined as apoptosis.

### RNA-seq assay

22Rv1 cells were treated with D45 (40 nM) and vehicle for 12 h, respectively. Total RNA was isolated with Trizol according to the manufacturer's instructions. After measurement by 5300 Bioanalyser (Agilent Technologies) and quantification by NanoDrop 2000 spectrophotometer (ThermoFisher Scientific), 1 μg RNA (OD_260/280_ = 1.8~2.2, OD_260/230_ ≥ 2.0, RQN ≥ 6.5, 28S:18S ≥ 1.0) was used to generate sequencing library for RNA-seq (Majorbio Biotech, China).

Differentially-expressed gene (DEG) analysis was performed using the DESeq2. Genes responsive to CDK9 degraders were identified based on a threshold of *P* < 0.05 and |Fold Change| ≥ 1.5. Gene Ontology (GO) and Kyoto Encyclopedia of Genes and Genomes (KEGG) pathway analyses were conducted using DAVID platform (https://david.ncifcrf.gov). Gene Set Enrichment Analysis (GSEA) was performed based on expression matrices (https://www.gsea-msigdb.org/gsea/msigdb/index.jsp). HRR genes were identified using Human DNA Repair Genes database (https://www.mdanderson.org). Data were visualized by bubble plots generated with the R package, and heatmaps, Venn diagrams, and GO/KEGG pathway visualizations were created using Bioinformatics online tools (http://www.bioinformatics.com.cn). For Gene Set Variation Analysis (GSVA), the WP_HR signaling pathway from MSigDB was analyzed using the GSVA R package, and sample-specific scores were generated for subsequent analysis. Drug sensitivity prediction was conducted using the oncoPredict R package. The training dataset was obtained from Genomics of Drug Sensitivity in Cancer (GDSC) database (https://www.cancerrxgene.org).

### Immunofluorescence staining

Cells were seeded in 6-well plates on coverslips at a density of 1 × 10^5^ cells/well and treated with D45, olaparib, or a combination for 12 h. For the knockdown experiment, after 24 h of siRNA treatment, olaparib was added for 12 h. Cells were fixed with ice-cold methanol, followed by blocking in 5% goat serum in the presence of 0.1% Triton X-100. The primary antibody was incubated at 4 ℃ overnight, and Alexa 488 or 555-conjugated secondary antibodies were incubated at room temperature for 1 h, respectively. Finally, the nuclei were stained with DAPI (P0131, Beyotime, China) and photographed under an Olympus FV3000 confocal laser scanning microscope.

### CUT&Tag (Cleavage Under Targets & Tagmentation) assay

C4-2 cells were treated with 40 nM D45 or vehicle for 12 h and harvested for CUT&Tag assay using NovoNGS CUT&Tag 4.0 High-Sensitivity Kit (N259-YH01, Novoprotein, China). In brief, 1 × 10^5^ cells were immobilized on concanavalin A beads and subsequently incubated with IgG or phospho-RNA Pol II CTD (C-terminal domain) (Ser2; 703108, ThermoFisher Scientific) as primary antibody for 2 h, and goat anti-rabbit (N269, Novoprotein) as the secondary antibody at room temperature for 1 h. These samples were then incubated with ChiTag transposon and fragmented with MgCl_2_. Genomic DNA was extracted and amplificated using the indexing primers. The enrichments on *BRCA1*, *BRCA2* and *RAD51* loci were measured by qPCR with the primers listed in Table S1.

### DR-GFP reporter assay

HRR efficiency was assessed using the DR-GFP reporter system 30, 31. Briefly, 293T cells were seeded into 6-well plates at a density of 2 × 10^5^ cells per well and cultured overnight. Cells were then co-transfected with the DR-GFP reporter plasmid and an I-SceI endonuclease expression plasmid using Lipofectamine 3000 transfection reagent (L3000015, ThermoFisher Scientific) according to the manufacturer's instructions. At 24 h post-transfection, cells were exposed to D45 and/or olaparib at the indicated concentrations for an additional 24 h. Subsequently, the proportion of GFP-expressing cells was quantified by flow cytometry (Agilent Technologies) and normalized to represent relative HRR efficiency.

### *Ex vivo* explants from human PCa specimens

The culture of human PCa explant *ex vivo* was performed as described previously 32, 33. Briefly, PCa tissues were cut into small fragments, put on the absorbable gelatin sponge (HSD-B, HUSHIDA Shanghai, China) and transferred into 6-well plates with 2 mL culture medium (DMEM/F-12 containing 10% FBS and penicillin plus streptomycin) per well at 37 °C in 5% CO_2_. The tissue fragments were treated with 20 nM D45 for 24 h, followed by 20 μM olaparib treatment for 48 h; treatments with vehicle or D45 alone (20 nM for 72 h) or olaparib alone (20 μM for 48 h) were used as controls. After treatment, tissue fragments were collected for histological assessment. Three PCa tissues were examined, including patient #1 (Gleason score 4+3/ISUP grade group 3), #2 (Gleason Score 4+3/ISUP grade group 3), and #3 (Gleason score 4+5/ISUP grade group 5).

### Statistical analyses

Statistical analyses were conducted using Prism 9.5.0 (GraphPad Software, USA). Continuous variables between two groups were compared by unpaired Student’s *t*-test. Multi-group comparisons were analyzed via one-way ANOVA (for one independent variable) or two-way ANOVA (for two independent variables), followed by Tukey’s post hoc test. Kaplan-Meier survival curves were generated, and intergroup differences were assessed by log-rank test. Pairwise correlations were quantified by Pearson correlation coefficient. *P* value < 0.05 was defined statistical significance.

## Results

### CDK9 overexpression in CRPC patients was associated with poor prognosis

**Figure 1:**
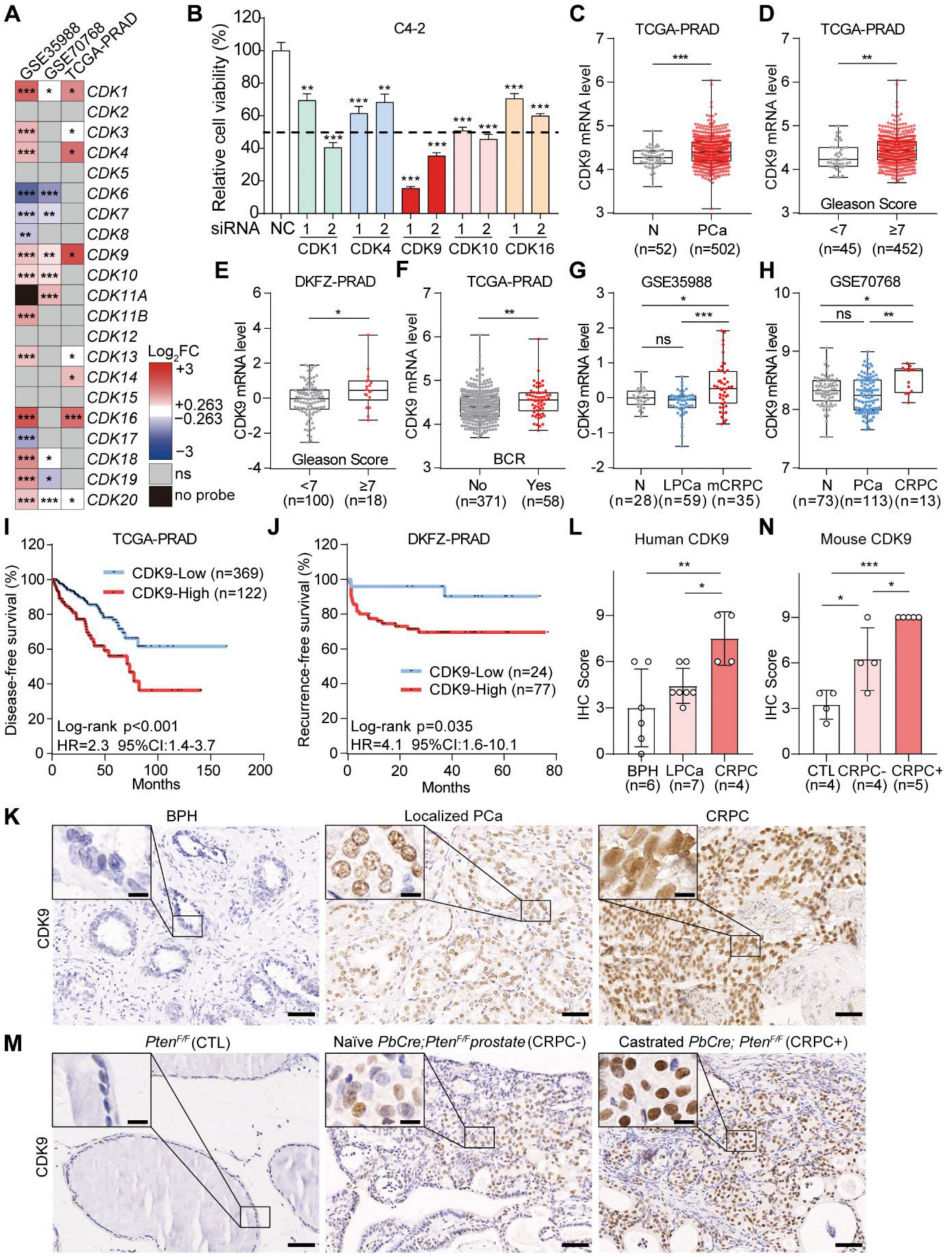
** CDK9 was overexpressed in CRPC. A,** The heatmap of CDK family members in three public PCa datasets. Color-coded blocks, the ratio of their expression levels between CRPC and non-CRPC (GSE35988 and GSE70768) or between BCR+ and BCR- PCa samples (TCGA-PRAD) with *P* < 0.05; gray blocks, *P* ≥ 0.05 (ns); black blocks, mRNA expression levels unavailable (no probe). *P* values were calculated with the Limma-moderated *t*-test. **B,** Cell viability of C4-2 cells transfected with the indicated siRNAs after 48 h by MTT assay. NC, siRNA control. Two siRNAs targeting different regions of *CDK* family genes were tested. **C,** *CDK9* mRNA levels in normal prostate and PCa tissues in TCGA-PRAD dataset. **D-E,** *CDK9* mRNA levels in PCa patients with different Gleason scores (< 7 and ≥ 7) in TCGA-PRAD (**D**) and DKFZ-PRAD (**E**) datasets. **F,** *CDK9* mRNA levels in PCa patients with and without BCR in TCGA-PRAD dataset. **G,** *CDK9* mRNA levels in normal, localized PCa (LPCa) and metastatic CRPC (mCRPC) samples in GSE35988 dataset. **H,** *CDK9* mRNA levels in normal, PCa and CRPC samples in GSE70768 dataset. **I-J,** The Kaplan–Meier plot of disease-free survival (**I**) and recurrence-free survival (**J**) in PCa patients, stratified by *CDK9* mRNA levels in TCGA-PRAD (**I**) and DKFZ-PRAD (**J**) datasets, respectively. **K-L,** IHC staining of CDK9 protein in human BPH, localized PCa (LPCa) and CRPC tissues from our cohort (**K**), with the quantification of CDK9 protein among three groups (**L**). **M-N,** IHC staining of CDK9 protein in aged matched *Pten^F/F^* prostate tissues (control, CTL), naïve *PbCre;Pten^F/F^* PCa (CRPC-) and castrated *PbCre;Pten^F/F^* PCa (CRPC+) (**M**), with the quantification of CDK9 protein among three groups (**N**). Scale bar, 50 μm and 10 μm (inset). Data were represented as mean ± SD. *P* values were determined by Student's *t*-test in **C-F**, and one-way ANOVA test in** B, G, H**, **L, N**. *P < 0.05, **P < 0.01, ***P < 0.001, ns, *P* ≥ 0.05.

To investigate whether CDK family member was correlated with CRPC, the integrated bioinformatic analyses were performed across three independent cohorts (TCGA-PRAD, GSE35988, and GSE70768) using the mRNA levels of 21 CDK members and the information on CRPC status or biochemical recurrence (BCR). CDK1, 4, 9, 10, and 16 were consistently upregulated in CRPC patients or PCa patients with BCR in at least two datasets (Figure 1A). In C4-2 CRPC cells, siRNA knockdown of these five CDKs (Figure S1A-E) revealed that depleting CDK9 decreased cell viability more potently than depleting the other four (Figure 1B). Moreover, CDK9 overexpression was observed in human PCa patients from TCGA-PRAD (*P* < 0.001; Figure 1C), which was significantly correlated with advanced Gleason score (*P* < 0.05; Figure 1D-E), T stage (*P* < 0.001; Figure S1F-G), metastatic progression (*P* < 0.01; Figure S1H-I) and recurred/progressed status (*P* < 0.001; Figure S1J) in TCGA-PRAD, DKFZ-PRAD, and GSE8511 datasets. Notably, PCa patients with BCR (TCGA-PRAD) or CRPC patients (GSE35988, GSE70768 and GSE74367) exhibited higher CDK9 expression levels than those without BCR (*P* < 0.01; Figure 1F) or non-CRPC patients (*P* < 0.05; Figure 1G-H and Figure S1K), portending poor patient survival (*P* < 0.05 in TCGA-PRAD and DKFZ-PRAD) (Figure 1I-J).

In line with the above results from public datasets, Western blotting data from our own cohort showed that both two isoforms of CDK9 proteins, due to different transcriptional start site 34, were significantly upregulated in PCa samples (n = 12), comparing with BPH samples (n = 12) (*P* < 0.001; Figure S1L-M). IHC staining further validated the marked overexpression of CDK9 protein in the nuclei of CRPC cells (n = 4), compared to naïve PCa (*P* < 0.05; n = 7); and BPH specimens (n = 6) showed the weakest CDK9 protein levels (Figure 1K-L). Murine *PbCre;Pten^F/F^* CRPC models recapitulated this CDK9 overexpression pattern, *i.e.*, weak expression in *Pten^F/F^* prostate epithelial cells, moderate in *PbCre;Pten^F/F^* PCa cells, and strong in *PbCre;Pten^F/F^* CRPC cells (*P* < 0.05; Figure 1M-N). Collectively, CDK9 was significantly associated with CRPC, indicating its role as a potential target for PCa aggressive progression.

### D45 decreased CDK9 protein levels in CRPC cells

**Figure 2:**
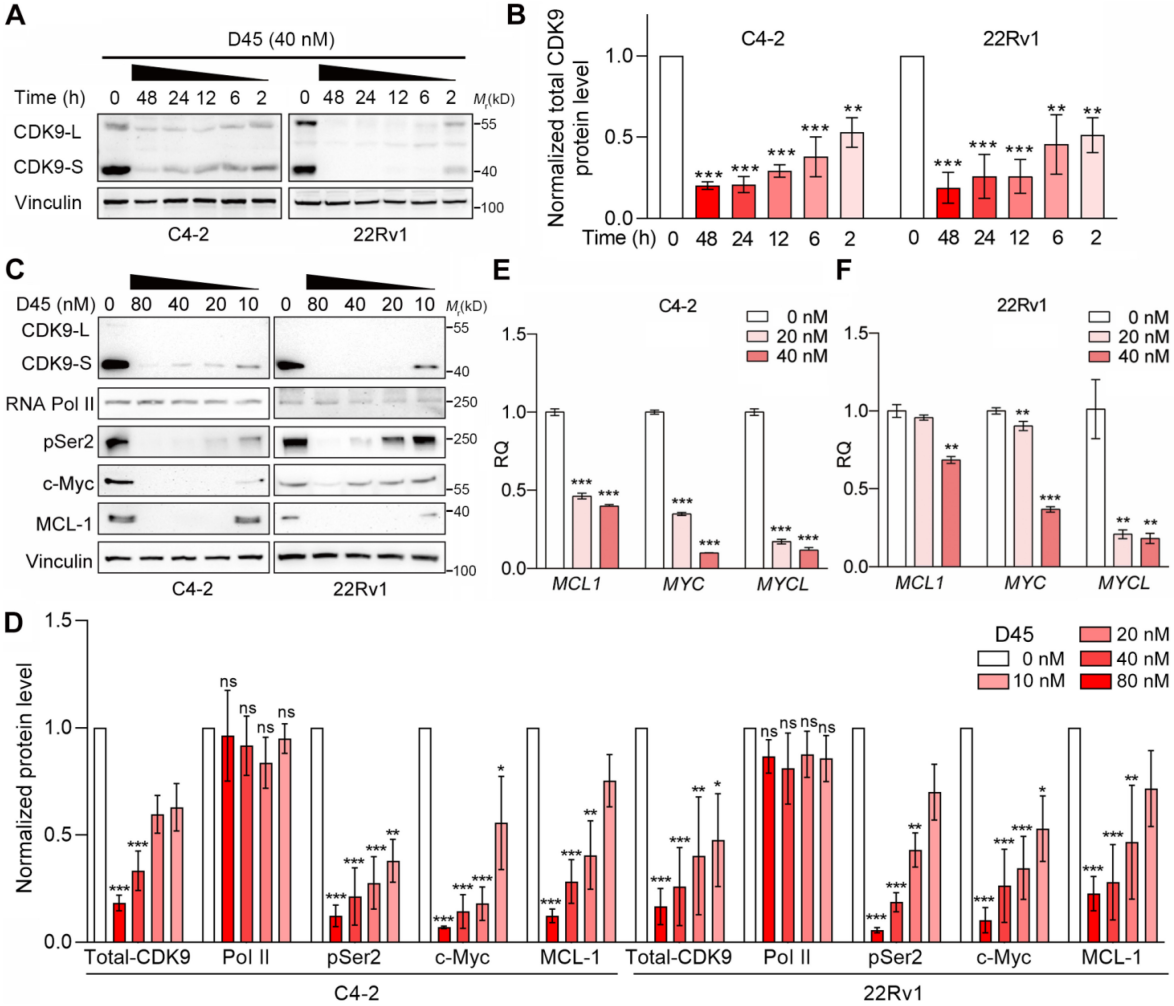
** CDK9 degradation by D45 treatment in CRPC cells. A-B,** Representative images (**A**) and quantification (**B**) for Western blots of CDK9 protein levels in C4-2 and 22Rv1 cells with 40 nM D45 at the indicated timepoints. CDK9-L, full-length isoform; CDK9-S, the short isoform. Total CDK9 protein levels (CDK9-L plus CDK9-S) were normalized by corresponding vinculin protein levels (n=3, mean ± SEM). **C-D,** Representative images (**C**) and quantification (**D**) for Western blots on protein levels of CDK9 and its downstream targets in C4-2 and 22Rv1 cells treated with various concentrations of D45 for 8 h. Vinculin was used as the normalization control (n=3, mean ± SEM). **E-F,** The mRNA levels of CDK9’s target genes in CRPC cells treated with D45 (**E**, C4-2 for 6 h; **F**, 22Rv1 for 12 h) by qRT-PCR assay (n=3, mean ± SD). *P* values determined by one-way ANOVA test in **B**, **D**, **E**, **F**. *P < 0.05, **P < 0.01, ***P < 0.001, ns, *P* ≥ 0.05.

Compound D45, a PROTAC previously shown to induce CDK9 degradation and suppress xenograft growth in triple-negative breast cancer 28, was evaluated for CDK9-targeting efficacy in two CRPC cell lines, C4-2 and 22Rv1 35. Concomitant degradation of both CDK9 isoforms, the full-length CDK9-L and the shorter CDK9-S, was induced by D45 in a time- and dose-dependent manner (Figure 2A-D). CDK9 ablation by D45 could suppress the phosphorylation of RNA Pol II at serine 2 (pSer2) - a hallmark of transcriptional elongation - concomitant with the reduced expression of two canonical CDK9 downstream targets (*MYC* and *MCL1*) at both transcriptional and protein levels. Another *MYC*-related gene, *MYCL*, was also downregulated at mRNA level by D45 treatment (Figure 2C-F). These findings showed that D45 was a potent CDK9 degrader in CRPC contexts, effectively disrupting CDK9-mediated transcriptional addiction via suppression of its downstream oncogenic networks.

### D45 decreased the viability of CRPC cells *in vitro*

**Figure 3:**
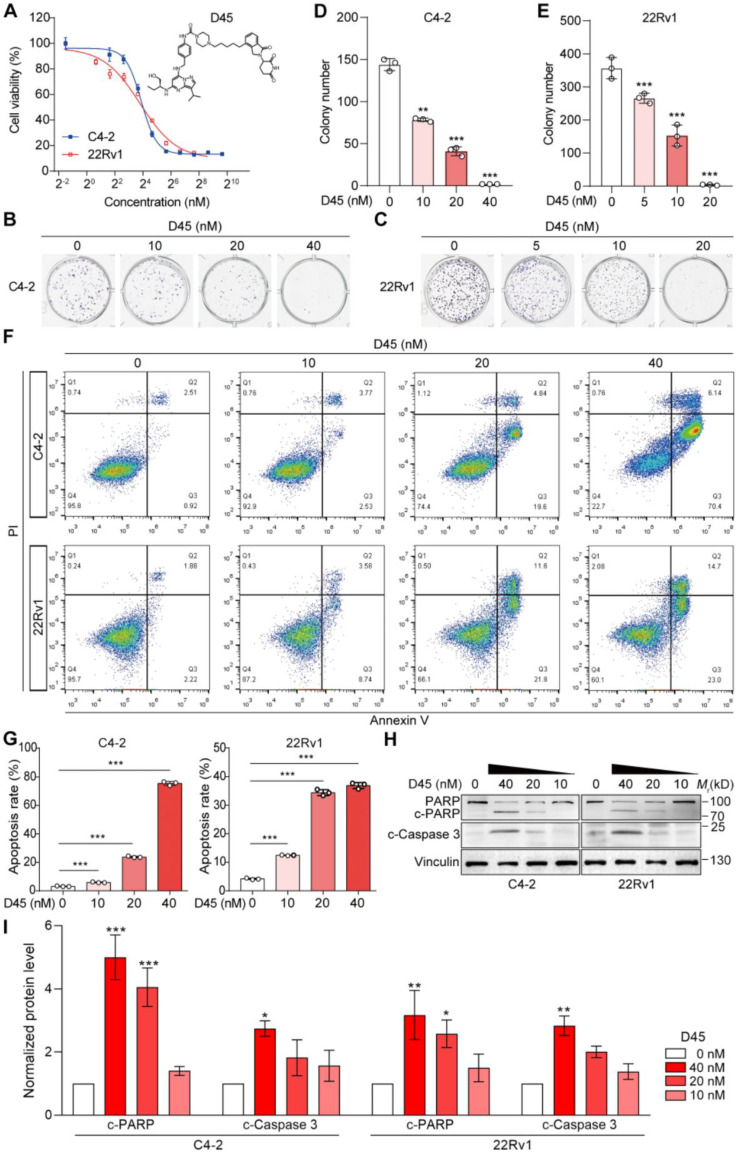
** D45 decreased the viability of CRPC cells *in vitro*. A,** Cell viability of C4-2 and 22Rv1 cells treated with D45 at the indicated concentrations for 72 h by MTT assay.** B-C,** The colony formation capability of C4-2 (**B**) and 22Rv1 cells (**C**) treated with D45 at the indicated concentration for 7 days. **D-E,** Quantification of the colony numbers in **B** and **C**, respectively. **F,** Apoptosis in C4-2 and 22Rv1 cells treated with D45 at the indicated concentrations for 72 h with the Annexin V/PI staining by flow cytometry. **G,** Quantification for the apoptosis percentage in C4-2 and 22Rv1 cells in **F**. **H-I,** Representative images (**H**) and quantification (**I**) for Western blots of cleaved Caspase 3 (c-Caspase 3) and cleaved PARP (c-PARP) proteins in C4-2 and 22Rv1 cells treated with D45 at the indicated concentrations for 72 h (n=3). Vinculin was used as the normalization control. Data were represented as mean ± SD in **D, E, G** or mean ± SEM in **I**. *P* values were determined by one-way ANOVA test in **D, E, G, I**. *P < 0.05, **P < 0.01, ***P < 0.001.

To assess the cytotoxic effects of D45 on CRPC cells, we conducted cell viability and colony formation assays using C4-2 and 22Rv1 cells. The results showed that both CRPC cells were sensitive to D45, with IC_50_ values of 14.70 nM for C4-2 and 13.99 nM for 22Rv1 (Figure 3A). D45 inhibited the colony formation capability of C4-2 and 22Rv1 cells in a dose-dependent manner (*P* < 0.01; Figure 3B-E). Flow cytometry analyses confirmed the dose-responsive apoptosis in CRPC cells to D45 (*P* < 0.001; Figure 3F-G), corroborated by cleavages of Caspase 3 and PARP, two apoptotic markers (Figure 3H-I). These results indicated that D45 effectively decreased the cell viability and enhanced apoptosis in CRPC cells.

### D45 inhibited the HRR gene expression in CRPC cells

**Figure 4:**
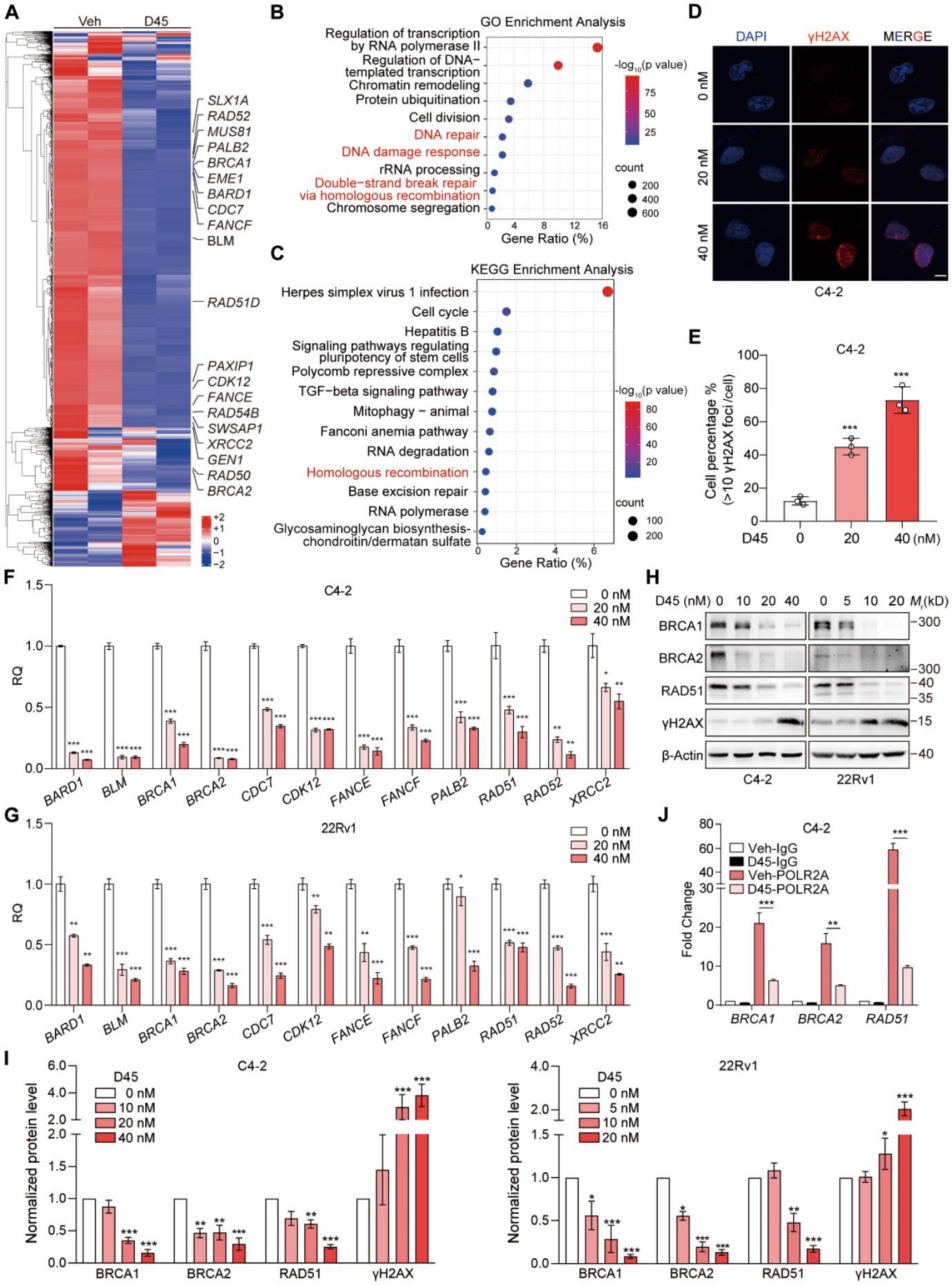
** Targeting CDK9 by D45 inhibited HRR gene expression in CRPC cells. A,** Heatmap depicting the DEGs with |Fold Change| ≥ 1.5 in 22Rv1 cells following 12 h-treatment with 40 nM D45 or vehicle (Veh) by RNA-seq. **B-C,** GO (**B**) and KEGG (**C**) enrichment analyses of DEGs from RNA-seq data in (**A**). **D-E,** The representative images of γH2AX foci in C4-2 cells treated with D45 for 12 h by immunofluorescence staining (**D**) and the percentage of cells that had more than 10 γH2AX foci per cell (**E**). DAPI was used for nuclei staining. Scale bar, 10 μm. **F-G,** mRNA levels of HRR genes in C4-2 (**F**) and 22Rv1 cells (**G**) treated with D45 at the indicated concentrations by qRT-PCR assay. **H-I,** Representative images (**H**) and quantification (**I**) for Western blots of HRR signaling proteins in C4-2 and 22Rv1 cells treated with D45 at the indicated concentrations (n=3). β-Actin was used as the normalization control. **J,** CUT&Tag-qPCR analysis of the indicated HRR genes by phospho-RNA Pol II CTD (Ser2) antibody (POLR2A) in C4-2 cells treated with 40 nM D45 or vehicle (Veh). IgG was used as a negative control. Data were represented as mean ± SD (in **E, F, G, J**) or mean ± SEM (in **I**). *P* values were determined by Hypergeometric test in **B, C**, by one-way ANOVA test in **E, F, G, I,** and Student's *t*-test in **J**. *P < 0.05, **P < 0.01, ***P < 0.001.

To delineate the mechanism underlying D45-induced cytotoxicity, transcriptomic profiling of D45-treated 22Rv1 cells (40 nM *vs* vehicle) was performed to reveal the CDK9-dependent transcriptional regulation network (GSE288031). Transcriptomic analysis detected a total of 41,016 gene transcripts. Among these, 4,283 genes were downregulated (10.4%) and 4,052 were upregulated (9.9%), while the majority (32,681 genes) showed no significant change. These data indicated that CDK9 degradation did not induce global transcriptional suppression. GO and KEGG analyses on DEGs demonstrated the coordinated suppression of DNA repair, double-strand break repair, and homologous recombination, which was regulated by HRR signaling (such as *BRCA1/2*, *FANCF*, *PALB2*,* RAD51D*, and *XRCC2*; Figure 4A-C), a pattern conserved across different datasets involving CDK9 inhibitors/degraders against various PCa cells (degrader LL-K9-3 to 22Rv1 cells in GSE205363 24; inhibitor AT7519 to LNCaP cells in GSE169090 36 and inhibitor CDKI-73 to LNCaP cells in GSE244716 37; Figure S2A-B), as well as breast cancer cells (degrader compound 28 to MDA-MB-231 cells in GSE282602 28; Figure S2C-D). In addition, GSVA on the human PCa dataset containing CRPC status information (GSE35988, n = 94) linked the elevated *CDK9* expression to HRR activation, including the significant upregulation of *BLM*, *BRCA1*, *FANCE*, *RAD52*, and* XRCC2*, along with the increase of WP_Score for HRR signaling pathway (r > 0.2, *P* < 0.05, 95% Confidence Interval (CI): 0.03-0.41; Figure S3).

Functional validation showed that D45 could induce γH2AX foci (DNA damage markers; Figure 4D-E), and decrease the mRNA (Figure 4F-G) and protein levels (Figure 4H-I) of HRR-related genes in C4-2 and 22Rv1 cells in a dose-dependent manner. Inhibiting CDK9 with siRNAs mimicked these results (Figure S4A-C). The CTD of human RNA Polymerase II Subunit A (POLR2A) comprises 52 tandem consensus heptapeptide repeats (Tyr1-Ser2-Pro3-Thr4-Ser5-Pro6-Ser7). pSer2 within these repeats is primarily regulated by CDK9 to facilitate transcriptional elongation 18, 38. By analyzing public proteomics datasets, we confirmed that pSer2 levels were significantly elevated at multiple positions in human PCas compared to corresponding normal prostate tissues (Figure S5A). Furthermore, mean pSer2 levels were positively correlated with both CDK9 protein abundance (r = 0.63, *P* < 0.001, 95% CI: 0.43-0.77 in OEP004998 dataset 39; r = 0.21, *P* = 0.0035, 95% CI: 0.069-0.33 in PXD049446 dataset 40) and downstream HRR-related proteins, such as RAD50 (r = 0.50, *P* = 0.0002, 95% CI: 0.26-0.59 in OEP004998 dataset; r = 0.57, *P* < 0.0001, 95% CI: 0.48-0.66 in PXD049446 dataset) (Figure S5B). CUT&Tag assay was thus performed 41, revealing that D45 inhibited the enrichment of pSer2-RNA Pol II at the ends of key HRR genes, including *BRCA1/2* and *RAD51* (Figure 4J). These findings suggested that the CDK9/pSer2 axis drove HRR activation by enhancing transcriptional elongation of essential repair genes, indicating CDK9 as the potential target to generate BRCAness.

### D45 synergized with olaparib to decrease CRPC cell viability and enhance DNA damage

Considering that targeting CDK9 downregulated a battery of HRR genes, including *BRCA1/2* and *RAD51*, and exhibited BRCAness, drug sensitivity analysis was performed in GSE35988 and found the predicted IC_50_ values of olaparib were significantly higher in CRPC patients than those in non-CRPC PCa (*P* < 0.001; Figure S6A). Those values were also positively correlated with CDK9 mRNA levels (*P* < 0.01; Figure S6B). The combination of CDK9 depletion and PARP inhibition was thus suggested for CRPC treatment.

**Figure 5:**
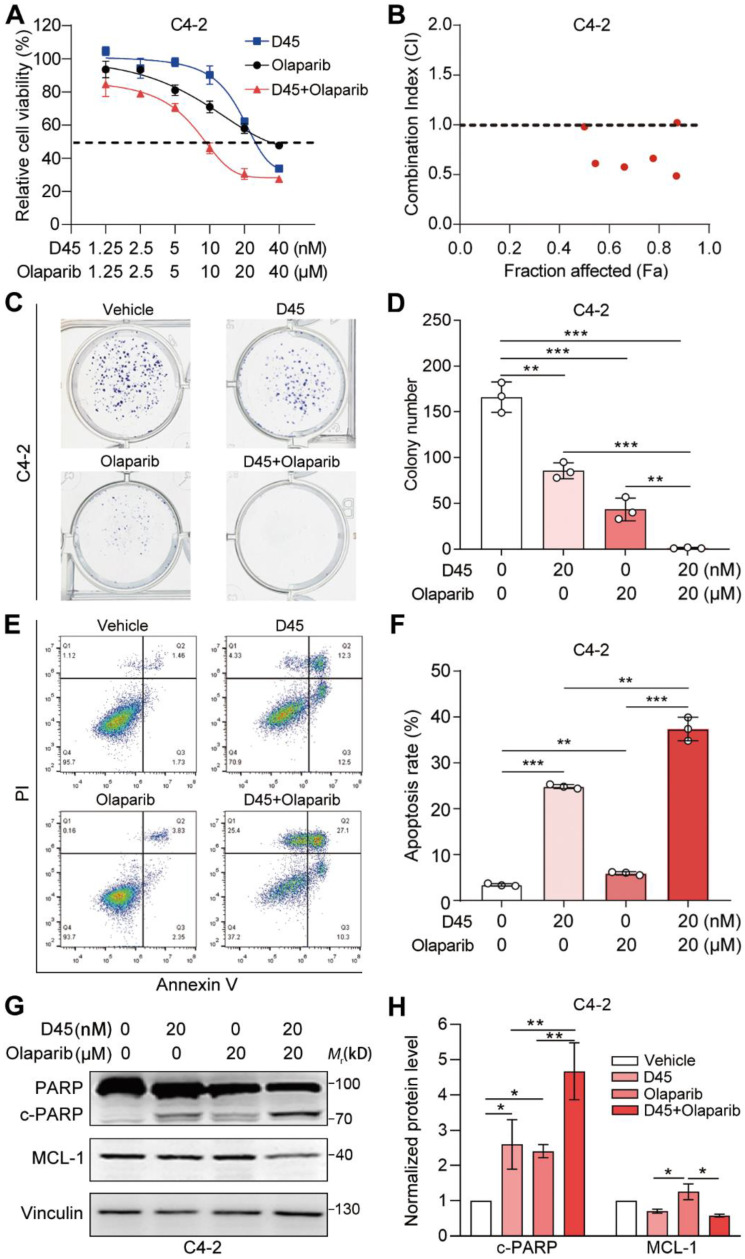
** D45 synergized with olaparib to decrease CRPC cell viability in C4-2 cells. A,** Cell viability of C4-2 cells treated with D45 and olaparib, alone or combined, at the indicated concentrations for 72 h by MTT assay.** B,** Combination index for D45 and olaparib in C4-2 cells by Chou-Talalay analysis.** C,** Colony formation capability of C4-2 cells treated with D45 and olaparib, alone or combined, at the indicated concentrations.** D,** Quantification of colony numbers in **C**.** E,** Apoptosis in C4-2 cells treated with D45 and olaparib, alone or combined, at the indicated concentrations for 72 h with the Annexin V/PI staining by flow cytometry. **F,** Quantification for the apoptosis percentage in **E**. **G-H,** Representative images (**G**) and quantification (**H**) for Western blots of cleaved PARP (c-PARP) and MCL-1 protein in C4-2 cells treated with D45 and olaparib, alone or combined, at the indicated concentrations for 72 h. Vinculin was used as the normalization control (n=3). Data were represented as mean ± SD in **D, F**, or mean ± SEM in **H**. *P* values were determined by two-way ANOVA test in **D, F,** and by one-way ANOVA test in **H**. *P < 0.05, **P < 0.01, ***P < 0.001.

**Figure 6:**
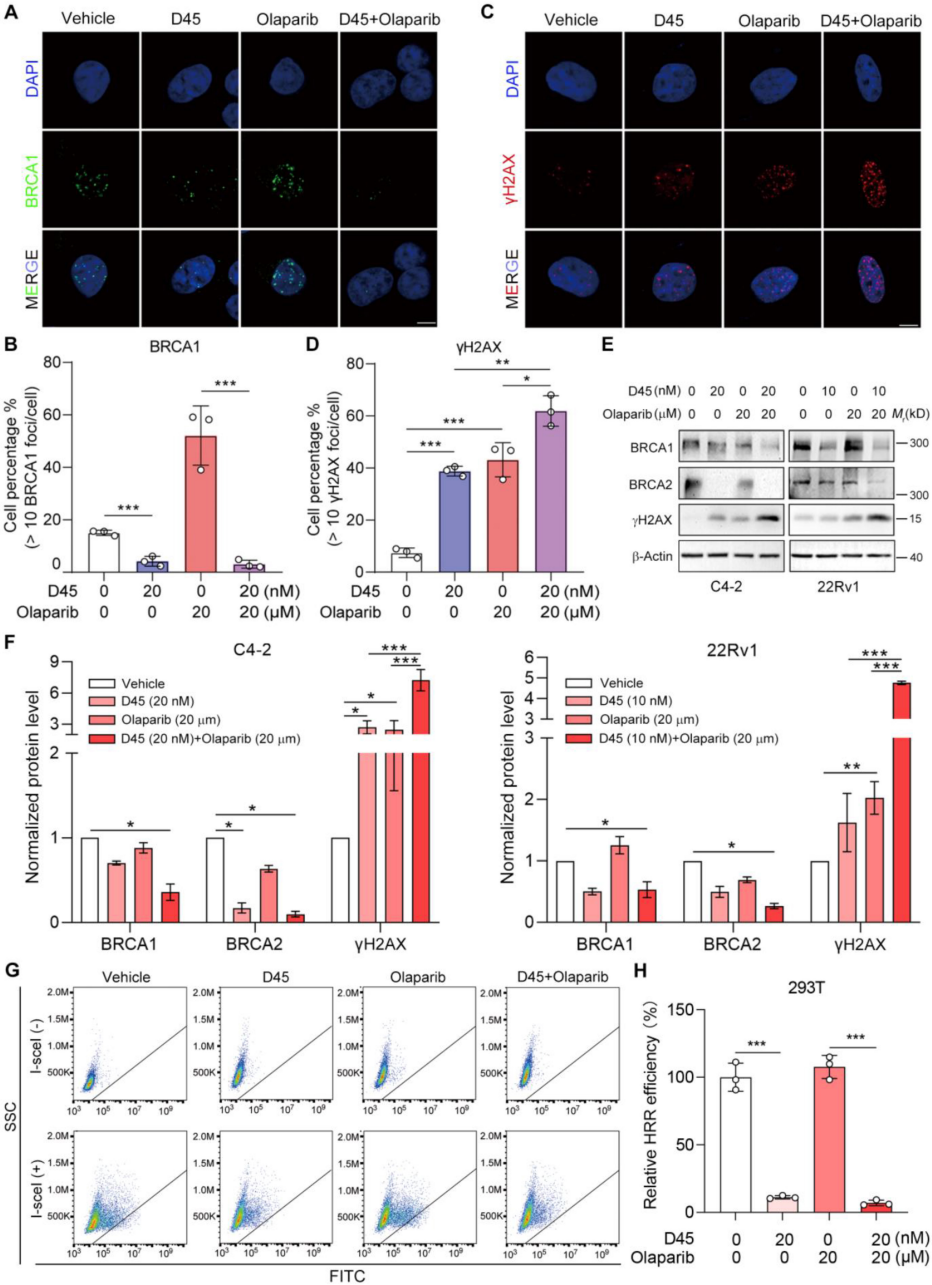
** D45 synergized with olaparib to enhance DNA damage. A, C,** Representative images of BRCA1 (**A**) and γH2AX foci (**C**) in C4-2 cells treated with D45 and olaparib, alone or combined, for 12 h by immunofluorescence staining. DAPI was used for nuclei staining. Scale bar, 10 μm. **B, D,** The percentage of cells that had more than 10 BRCA1 foci per cell in (**A**) and more than 10 γH2AX foci per cell in (**C**) (mean ± SD). **E-F,** Representative images (**E**) and quantification (**F**) for Western blots of BRCA1, BRCA2 and γH2AX proteins in C4-2 and 22Rv1 cells treated with D45 and olaparib, alone or combined, for 72 h (n=3, mean ± SEM). β-Actin was used as the normalization control. **G-H,** DR-GFP reporter assay evaluating the effects of D45 and olaparib on HRR efficiency in 293T cells (**G**). Relative HRR efficiency was normalized to the vehicle control (100%) (**H**). *P* values determined by two-way ANOVA test in **B, D, H,** and one-way ANOVA test in **F**. *P < 0.05, **P < 0.01, ***P < 0.001.

In C4-2 and 22Rv1 cells, olaparib alone decreased cell viability only to ~ 50% even at the highest concentration (40 μM for C4-2 and 80 μM for 22Rv1). In the presence of D45, olaparib (10 μM) plus D45 (10 nM) in C4-2 cells (Figure 5A) or plus D45 (5 nM) in 22Rv1 cells (Figure S7A) achieved > 50% inhibitory effects. In order to explore whether it is a synergistic effect, Chou-Talalay analysis was performed. The resulting CI values were less than 1 for most drug-combination groups (Figure 5B, Figure S7B), indicating CDK9 degrader-olaparib synergy in both cells. The dose combinations of D45: olaparib (20 nM: 20 μM) for C4-2 cells and D45: olaparib (10 nM: 20 μM) for 22Rv1 cells were therefore chosen for further studies. This drug combination significantly inhibited the colony formation capability (*P* < 0.001; Figure 5C-D, Figure S7C-D) and enhanced apoptosis (*P* < 0.01; Figure 5E-F, Figure S7E-F) in both cells. Western blotting assay confirmed the increased PARP cleavage and the decreased MCL-1 protein level (Figure 5G-H, Figure S7G-H). Additionally, this drug combination significantly reduced the BRCA1 foci (*P* < 0.001; Figure 6A-B) and elevated the γH2AX foci (*P* < 0.05; Figure 6C-D) by immunofluorescence staining in C4-2 cells. Western blotting assay verified the decrease of BRCA1/2 proteins and the increase of γH2AX protein in both C4-2 and 22Rv1 cells (Figure 6E-F). Moreover, the DR-GFP reporter assay indicated that D45, both as a monotherapy and in combination with olaparib, significantly impaired HRR efficiency (*P* < 0.001), whereas olaparib alone had no significant effect in 293T cells (Figure 6G-H). These effects were consistent with the observations using CDK9 siRNAs with olaparib (Figure S8A-D), further validating that decrease of CDK9 expression by either CDK9 degrader or CDK9 siRNA synergized with olaparib to exacerbate DNA damage by suppressing the HRR pathway.

### Sequential administration of D45 and olaparib significantly suppressed PCa xenograft growth *in vivo*

A 28-day repeated-dose toxicity study was conducted in C57BL/6 mice using the sequential dosing schedule (Figure S9A). Throughout the treatment period, no mortality or severe moribundity was observed, and body weight remained stable across all four groups (Figure S9B). Following the four-week administration, systemic safety was evaluated via CBC and serum biochemical analysis (Table S4). While the erythroid system exhibited moderate fluctuations, the most critical safety signal was a marked reduction in the leukocyte population. Specifically, white blood cell (WBC) and lymphocyte (LYMPH) counts decreased significantly in all three groups with drug treatment (*P* < 0.001); monocyte (MONO) and eosinophil (EO) counts were significantly reduced in the olaparib monotherapy (*P* < 0.05) and combination group (*P* < 0.01); a significant decline in neutrophil (NEUT) was observed exclusively in the combination group (*P* < 0.05). A myelosuppression was thus indicated, consistent with clinical observations of CDK9 inhibitor (dinaciclib) 42 and olaparib 43. Whereas, serum biochemical analysis revealed no significant alterations in hepatic (ALT and AST) or renal (CREA and urea) function markers. Histopathological evaluation via H&E staining of major organs, including the heart, liver, lung, kidney, small intestine, and spleen, confirmed no significant morphological damage across all groups (Figure S9C).

**Figure 7:**
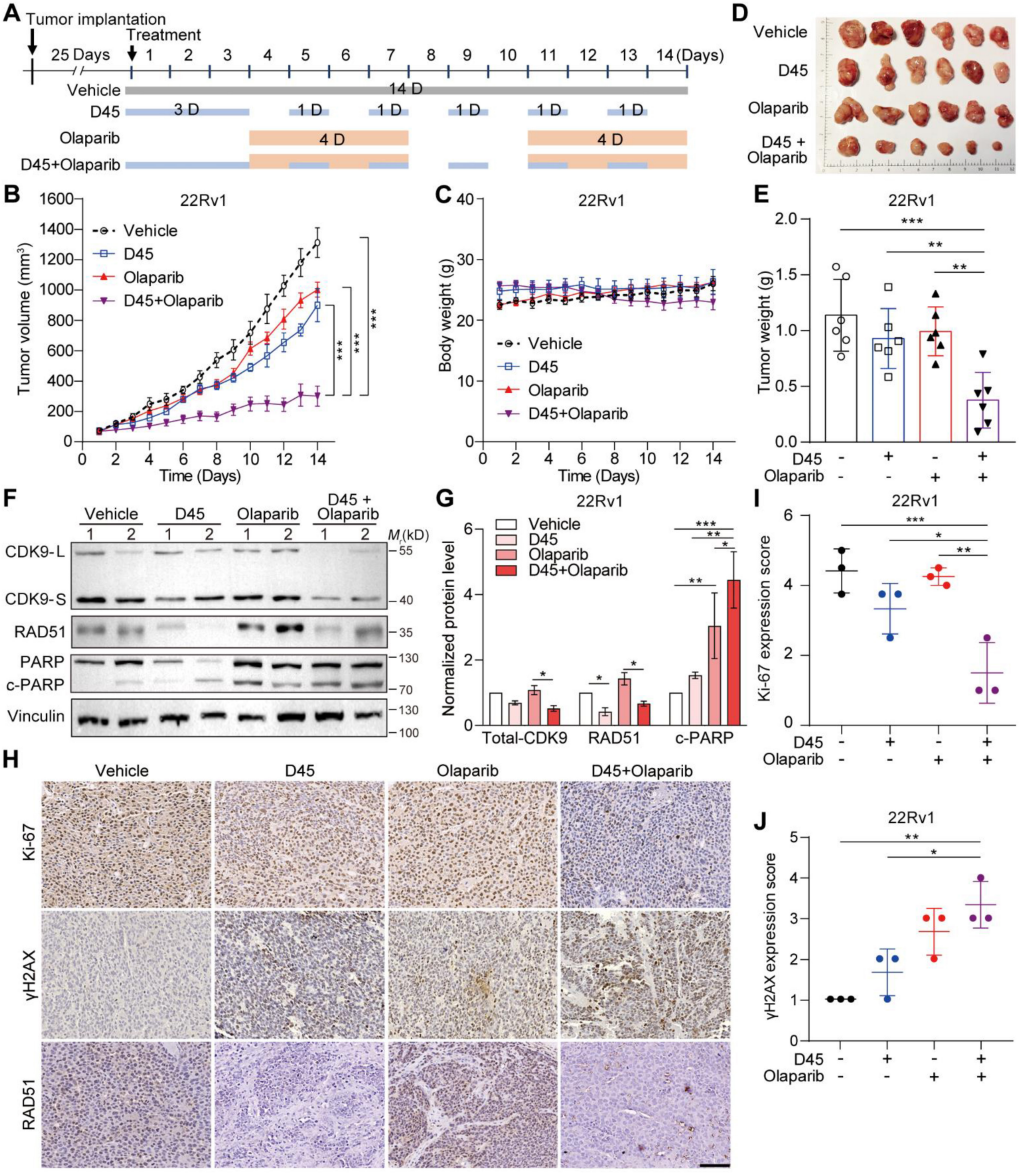
** Sequential administration of D45 and olaparib significantly suppressed 22Rv1 xenograft growth. A,** Experimental scheme of sequential regimen for D45 and olaparib, alone or combined, in 22Rv1 xenografts. The xenograft-bearing mice were divided into four groups: Vehicle (n = 6), D45 (1.5 mg/kg, n = 6), olaparib (50 mg/kg, n = 6), and D45 plus olaparib (1.5 mg/kg and 50 mg/kg, n = 6). **B-E,** Tumor growth curve (**B**), body weight curve (**C**), tumor photo (**D**), and tumor weight (**E**) of 22Rv1 xenografts treated with D45 and olaparib, alone or combined. **F-G,** Representative images (**F**) and quantification (**G**) for Western blots of CDK9, RAD51, and cleaved PARP proteins in 22Rv1 xenografts treated with D45 and olaparib, alone or combined (n=3). Vinculin was used as the normalization control. **H,** The representative IHC images of Ki-67, γH2AX and RAD51 proteins in 22Rv1 xenografts treated with D45 and olaparib, alone or combined. Scale bar, 50 μm. **I-J,** Quantification of Ki-67 (**I**) and γH2AX proteins (**J**) in 22Rv1 xenografts treated with D45 and olaparib, alone or combined. Data were represented as mean ± SD in **B-E, I-J**, or mean ± SEM in **G**. *P* values were determined by two-way ANOVA test in **B-E, I-J** and by one-way ANOVA test in **G**. *P < 0.05, **P < 0.01, ***P < 0.001.

Regarding the tolerable toxicity profile, we established the subcutaneous 22Rv1 xenografts in nude mice to evaluate the efficacy of D45-olaparib combination* in vivo*. We implemented a sequential regimen to induce BRCAness status prior to PARPi treatment (Figure 7A): Low-dose D45 priming (1.5 mg/kg, *ip*, *q.d.* for first three days) was administered to induce the initial BRCAness, followed by intermittent combination therapy with D45 (1.5 mg/kg, *ip*, every other day) and olaparib (50 mg/kg, *ip*, 4×/week). After two weeks of combined administration, we found that D45 or olaparib alone had little effects compared to the vehicle group; however, drug combination significantly suppressed the xenograft growth comparing to the single drug group (*P* < 0.001; Figure 7B), without significant change in body weight (Figure 7C). At the endpoint, xenografts were harvested and weighed to confirm the tumor growth suppression (*P* < 0.01; Figure 7D-E). Western blotting assay demonstrated that combination treatment decreased CDK9 and RAD51 protein levels and increased PARP protein cleavage in 22Rv1 xenografts (Figure 7F-G). Consistently, IHC staining revealed that the combination treatment remarkably reduced RAD51 and Ki-67 (cell proliferation marker) protein levels, and enhanced γH2AX protein expression in 22Rv1 xenografts (Figure 7H-J).

**Figure 8:**
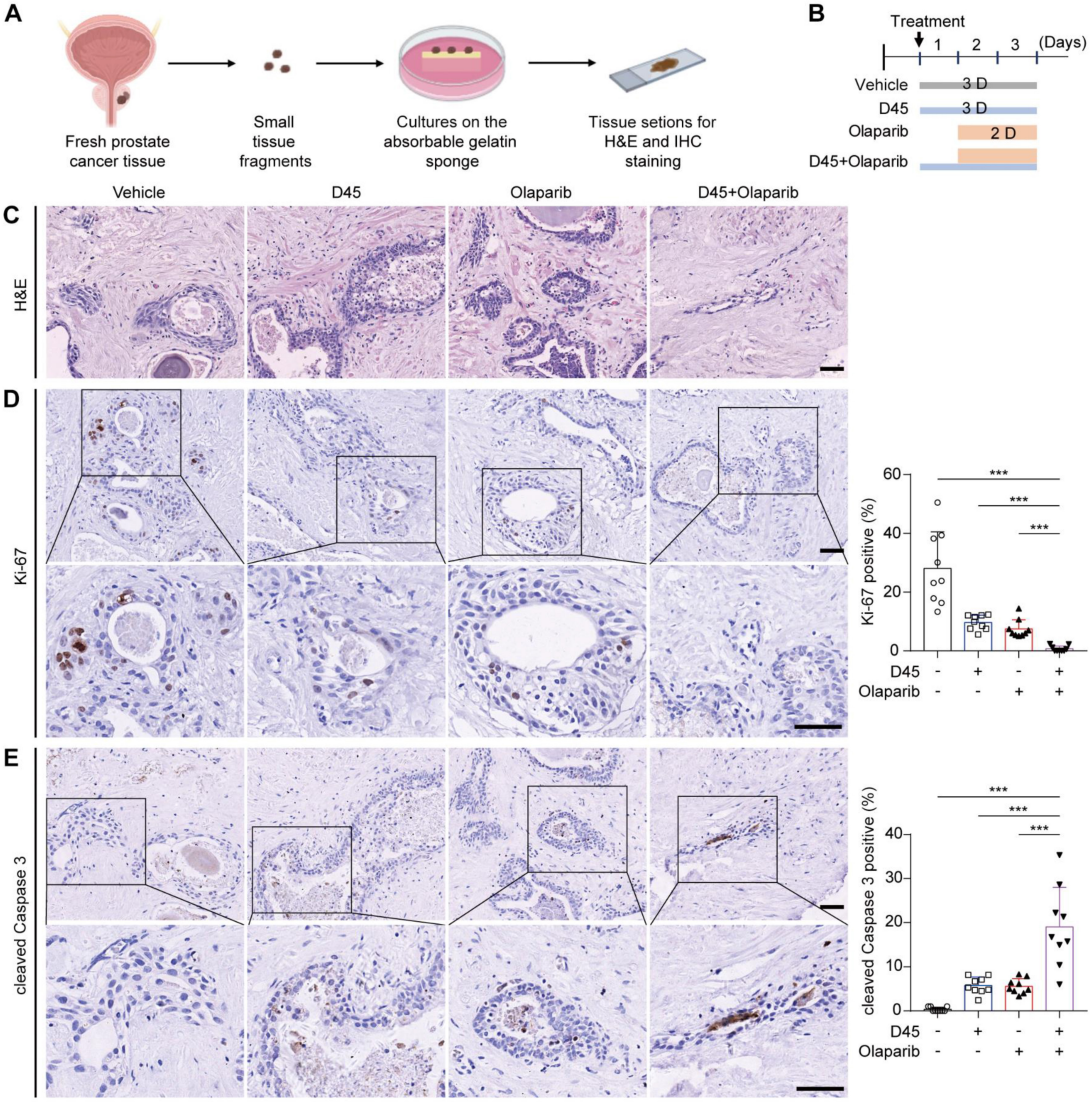
** Sequential treatment of D45 and olaparib significantly inhibited cell proliferation and induced apoptosis in human PCa explants *ex vivo*. A,** Schematic diagram of the PCa explant culture process. **B,** Experimental schematic of PCa explants treated with D45 and olaparib, alone or combined. The PCa explants were divided into four groups (n = 3/group): Vehicle, D45 (20 nM), olaparib (20 μM), and D45 plus olaparib (20 nM and 20 μM). **C,** H&E staining for PCa explants treated with D45 and olaparib, alone or combined. Scale bar, 50 μm. **D-E,** IHC images and quantification for Ki-67 (**D**) and cleaved Caspase 3 proteins (**E**) in PCa explants treated with D45 and olaparib, alone or combined. Scale bar, 50 μm and 25 μm (inset). Data were represented as mean ± SD with *P* values determined by two-way ANOVA test in **D, E**. ***P < 0.001.

The combination regimen was further validated using *ex vivo* explants from human PCa tissues (Figure 8A-C). IHC analysis revealed that the combination therapy group showed a significant decrease in Ki-67 expression (*P* < 0.001; Figure 8D) and a significant increase in cleaved Caspase 3 (*P* < 0.001; Figure 8E), compared to the vehicle group and the single drug group, respectively.

Taken together, the sequential treatment of D45-olaparib synergistically improved antitumor efficacy *in vivo* and *ex vivo* via suppressing HRR pathway, inducing DNA damage, and promoting apoptosis in PCa.

## Discussion

In this study, we identified that CDK9 expression level was significantly elevated in CRPC patients and served as a poor prognosis in PCa patients, based on the analyses of 921 PCa patients from 6 public datasets, followed by the validation in our own cohort. Moreover, a CDK9 selective degrader (D45) was proved to efficiently reduce CDK9 protein level, inhibit the transcription of HRR genes, and induce “BRCAness” in CRPC cells. Notably, a sequential, low-dose regimen of D45 and olaparib achieved synthetic lethality and overcame PARPi resistance with minimal toxicity.

The clinical success of PARPi has established a paradigm for synthetic lethality in cancer treatment, particularly in tumors harboring *BRCA1/2* mutations 44. However, the emergence of acquired resistance has significantly limited their long-term efficacy. To overcome these challenges and expand the utility of PARPi beyond patients with HRR-deficient tumors, combination therapeutic strategies have become imperative.

For BRCA1/2-deficient PCa, tumors can evade PARPi-induced synthetic lethality through various mechanisms, including the restoration of HRR functionality via secondary mutations in key HRR genes 45 and loss of *CHEK2* 46. To counteract this, rational combinations have been explored. For instance, combination with AR inhibitors in CRPC not only targeted the primary oncogenic driver but also modulated the expression of DNA damage repair genes, thereby preventing HRR recovery and re-sensitizing tumors to PARPi 47. However, BRCA1/2-deficient PCa patients are very limited because of the low mutation frequency. For BRCA1/2-proficient PCa (BRCAness-negative), a primary objective is to pharmacologically induce a BRCAness phenotype to confer their sensitivity to PARPi. This strategy involves exploiting vulnerabilities in PCa cells to create a synthetic lethal partnership with PARP inhibition. Promising approaches have been intensively explored, including targeting key nodes in the DNA damage response (DDR) network, such as ATR or depleting HRR-associated proteins (*e.g*. RAD51 and BRCA1/2). A multi-center Phase II study (NCT03787680) is testing the activity of olaparib and AZD6738 (an ATR inhibitor) in mCRPC (https://clinicaltrials.gov/study/NCT03787680). In addition, a peptide-based PROTAC drug targeting BRCA2, combined with PARP inhibitors to induce a synthetic lethal state, promoted cell death and induced tumor regression in PCa models 48. In addition, targeting cell cycle regulators, such as CDK4 and CDK6 by CDK4/6i (palbociclib or abemaciclib) shows synergistic anti-CRPC cell viability with PARPi (olaparib) 49.

The combination of a CDK9 selective degrader (D45) and PARPi (olaparib) was investigated in CRPC models *in vitro* and *in vivo*. CDK9 inhibition or degradation alone has been demonstrated to suppress multiple oncogenic transcriptional and epigenetic pathways in PCa 37, 50, suggesting a potential therapeutic advantage over traditional treatments, particularly in patients with advanced disease 51. However, first-generation CDK9 inhibitors operate through an occupancy-driven model by competing for the ATP-binding pocket to block kinase activity, which requires high drug concentrations to achieve efficacy and lacks sufficient selectivity, resulting in severe on- and off-target systemic toxicity that limits clinical applicability 20. In contrast, CDK9 degraders operate via an event-driven (catalytic) mechanism, leveraging E3 ubiquitin ligase recruitment to induce the proteasomal degradation of the target protein. Beyond merely neutralizing enzymatic activity, we further addressed the critical non-kinase functions of CDK9. As a pivotal scaffolding protein, CDK9 maintains the structural integrity of the P-TEFb complex and other transcriptional regulatory assemblies 52. In addition, CDK9 forms a specialized complex with Cyclin K (rather than Cyclin T1) which localizes to chromatin during replication stress. This complex is essential for stabilizing stalled replication forks and limiting the accumulation of single-stranded DNA, thereby facilitating recovery from replication arrest 53. Therefore, considering that CDK9 degraders could comprehensively abrogate the protein’s enzymatic activity, scaffolding roles, and genome-protective mechanisms, we explored whether CDK9 degradation could serve as a combination partner with PARPi in CRPC models.

Our novel combination strategy included: 1) a CDK9 PROTAC (D45) was chosen for its high potency and high selectivity: It was shown that the IC_50_ value of D45 was 13.6 nM in MDA-MB-231 cells and at a concentration of 10 nM, D45 selectively degraded CDK9 without affecting CDK1, 2, 4, 5, 6 and 7 proteins 28. We also confirmed its high potency as IC_50_ values below 15 nM in both CRPC cell lines. 2) the synergistic effect between D45 and olaparib was demonstrated: the CI values were less than 1 at most drug-combination groups in CRPC cells by Chou-Talalay analysis. D45-olaparib synergy allowed for the use of substantially lower, more therapeutically tolerable doses of each agent. For example, our dosing regimen of drug combination was D45 (1.5 mg/kg every other day) and olaparib (50 mg/kg four consecutive days each week) for two weeks. The growth of 22Rv1 xenografts was significantly suppressed, compared to vehicle or single agent (*P* < 0.001), without apparent body weight loss. When D45 alone was administered in MDA-MB-231 xenografts, the dosage (2.5 mg/kg every day for one week) resulted in a significant body weight loss at the 7^th^ day (*P* < 0.01) 28. Therefore, this dose-sparing effect is of critical importance, as it promises to mitigate toxicity profiles while maintaining robust anticancer efficacy and enhancing the potential therapeutic window for CDK9-based therapies. 3) the CDK9-PARP combination pair was demonstrated: D45-olaparib synergy was rooted in a logically sequential mode of action *in vivo*, *i.e.* D45 first acted to pharmacologically induce a BRCAness state in BRCA1/2-proficient CRPC cells, effectively creating an acquired vulnerability; olaparib then exploited this induced vulnerability by imposing synthetic lethality. This was a fundamental advance beyond the single-agent mechanism of CDK9 degrader, which primarily relies on transcriptional inhibition and the subsequent induction of apoptosis. This drug combination activated a second, powerful cytotoxic pathway: the pre-existing DNA damage caused by CDK9 degrader was exacerbated by PARP inhibition, leading to the accumulation of unrepaired DNA double-strand breaks, genomic instability and ultimate cell death. This dual-pathway activation accounted for the enhanced suppression of CRPC cell growth and apoptosis induction observed in our models. However, the clinical translation of CDK9-PARP targeting regimen must anticipate potential resistance landscapes. Beyond canonical PARPi resistance mechanisms, such as HRR restoration and SLFN11 loss 54, 55, and CDK9-specific adaptations including the L156F mutation 56 or ABCB1-mediated efflux 57, synergistic resistance may emerge through compensatory bypass signaling or epigenetic remodeling. For instance, cells might upregulate alternative transcriptional kinases (*e.g.*, CDK12) or shift their survival reliance to the ATR-CHK1 axis to stabilize compromised replication forks; and epigenetic plasticity induced by CDK9 suppression could enable adaptive transcriptional rewiring. Deciphering these adaptive trajectories will be pivotal for designing intermittent dosing regimens to delay resistance onset or developing next-generation polytherapies to circumvent acquired resistance.

Unlike traditional transcriptional inhibitors, which are frequently constrained by short half-lives and the requirement for a high maximum concentration (Cmax) to maintain target occupancy, D45 exhibited a favorable pharmacokinetic profile with a prolonged half-life (17.34 h) and substantial systemic exposure (area under the plasma concentration-time curve (AUC) of 9,649 h·ng/mL following a 5 mg/kg *i.v.* dose) in mice 28. Inherent interspecies differences necessitate rigorous Phase I dose-escalation studies to establish the optimal clinical exposure-response relationship in humans. Moreover, a four-week repeated-dose toxicity study evaluating our sequential dosing schedule revealed no mortality or moribundity, with body weights remaining stable across all experimental cohorts. While CBC, serum biochemical, and histopathological analyses confirmed the absence of significant structural damage to major organs, we observed moderate hematologic toxicity in the CDK9 degrader, olaparib, and combination groups, which was consistent with previous reports for these drug classes 42, 43. Future optimization might prioritize refining intermittent dosing schedules to fully leverage the sustained pharmacodynamic effects of protein degradation while maximizing the therapeutic window.

## Conclusions

CDK9 depletion represents a promising strategy to induce BRCAness and to expand the therapeutic use of PARPi in CRPC patients without HRR-gene mutations. Moving beyond the conventional use of CDK9 depletion as a standalone transcriptional blocker, we re-define it as a potent “sensitizing agent” that reprograms DNA repair capability of CRPC cells. Our findings advocate for a strategic shift in the use of CDK9-targeting compounds, highlighting their greatest potential not as monotherapies, but as rational partners in combination regimens designed to induce synthetic lethality and overcome the inherent limitations of PARPi in HRR-proficient cancers.

## Supplementary Material

Supplementary figures and tables.

## Data Availability

The RNA-seq data were available in GEO database with the number GSE288031. Other data that support the findings of this study are available from the corresponding author upon reasonable request.

## Abbreviations

PCa: Prostate cancer
CRPC: Castration-resistant prostate cancer
AR: Androgen receptor
mCRPC: Metastatic CRPC
HRR: Homologous recombination repair
PARP: Poly (ADP-ribose) polymerase
PARPi: PARP inhibitor
CDK: Cyclin-dependent kinase
RNA Pol II: RNA polymerase II
PROTAC: Proteolysis targeting chimera
TCGA: The Cancer Genome Atlas
GEO: Gene Expression Omnibus
BPH: Benign prostatic hyperplasia
siRNA: Small interfering RNA
IACUC: Institutional Animal Care and Use Committee
*ip*: Intraperitoneally
CBC: Complete blood count
ALT: Alanine aminotransferase
AST: Aspartate aminotransferase
CREA: Creatinine
H&E: Hematoxylin and eosin
DAB: 3,3'-diaminobenzidine
IHC: Immunohistochemistry
RT-qPCR: Reverse transcription-quantitative polymerase chain reaction
CI: Combination index
PI: Propidium iodide
DEG: Differentially-expressed gene
GO: Gene Ontology
KEGG: Kyoto Encyclopedia of Genes and Genomes
GSEA: Gene Set Enrichment Analysis
GSVA: Gene Set Variation Analysis
GDSC: Genomics of Drug Sensitivity in Cancer
CUT&Tag: Cleavage Under Targets & Tagmentation
CTD: C-terminal domain
BCR: Biochemical recurrence
pSer2: Phosphorylation of RNA Pol II at serine 2
95% CI: 95% Confidence Interval
POLR2A: RNA Polymerase II Subunit A
WBC: White blood cell
LYMPH: Lymphocyte
MONO: Monocyte
EO: Eosinophil
NEUT: Neutrophil
DDR: DNA damage response
Cmax: maximum concentration
AUC: Area under the plasma concentration-time curve
