# Supplementary material for: CDK9 degrader induces BRCAness and sensitizes castration-resistant prostate cancer to PARP inhibitor
Source: Theranostics. 2026 Jun 17;16(13):7495–513. doi: 10.7150/thno.131907 (PMC13295117; doi:10.7150/thno.131907)
Supplement: Supplementary file 1 — Supplementary figures and tables. [file thnov16p7495s1.pdf]

Supplementary Information

CDK9 degrader induces BRCAness and sensitizes castration-resistant prostate cancer to PARP inhibitor

Jiaxuan Li[#], Jingya Sun[#], Weisong Tan[#], Guoqi Li, Wenjie Xiao, Jiajun Wu, Wei Yang, Changqing Chen, Yang Li, Jiakuan Liu, Yuanyu Liu, Dong Liu, Xiao-hua Chen, Rujian Zhu[*], Ruimin Huang[*], Jun Yan[*]

[#]These authors contributed equally to this article.

[*]**Corresponding authors:**

Jun Yan, Ph.D., Fudan University, Shanghai 200032, China. Phone: 86-21-54237454; E-mail: yan_jun@fudan.edu.cn

Ruimin Huang, Ph.D., Shanghai Institute of Materia Medica, Chinese Academy of Sciences, Shanghai 201203, China. Phone: 86-21-68077978; E-mail: rmhuang@simm.ac.cn

Rujian Zhu, M.D., Shanghai Pudong Hospital, Fudan University Pudong Medical Center, Shanghai, China. Phone: 86-18918753975; E-mail: tzzhurj@163.com

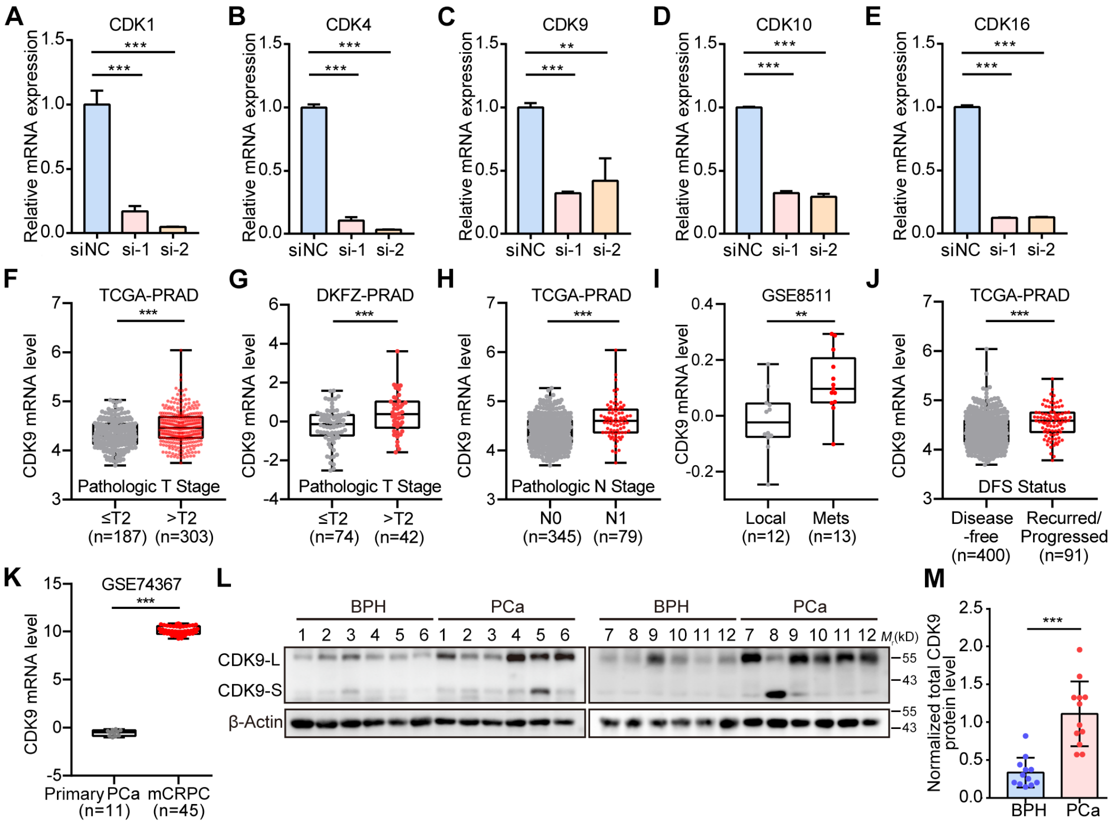

Supplementary Figure S1. The expression of CDK family members in human PCa cells and patients. A-E, Validation of knockdown efficiency using siRNAs targeting *CDK1* (**A**), *CDK4* (**B**), *CDK9* (**C**), *CDK10* (**D**), and *CDK16* (**E**) in C4-2 cells. NC, siRNA control. Two siRNAs (si-1 and si-2) targeting different regions of CDK family genes were tested. **F-G,** *CDK9* mRNA levels in PCa patients with different tumor stages (≤T2 and >T2) from TCGA-PRAD (**F**) and DKFZ-PRAD (**G**) datasets. **H,** *CDK9* mRNA levels in PCa patients with different N stages (N0 and N1) from TCGA-PRAD dataset. **I,** *CDK9* mRNA levels in local and metastatic (Mets) PCa tissues from GSE8511 dataset. **J,** *CDK9* mRNA levels in disease-free and recurred/progressed PCa samples from TCGA-PRAD dataset. **K,** *CDK9* mRNA levels in primary PCa and mCRPC samples from GSE74367 dataset. **L-M,** Representative images (**L**) and quantification (**M**) for Western blots of CDK9 protein levels in human BPH and PCa tissues from our cohort. β-Actin was used as the normalization control. Data were represented as mean ± SD. *P* values were determined by one-way ANOVA test in **A-E**, and Student's *t*-test in **F-K, M**. $^{**}P < 0.01$; $^{***}P < 0.001$.

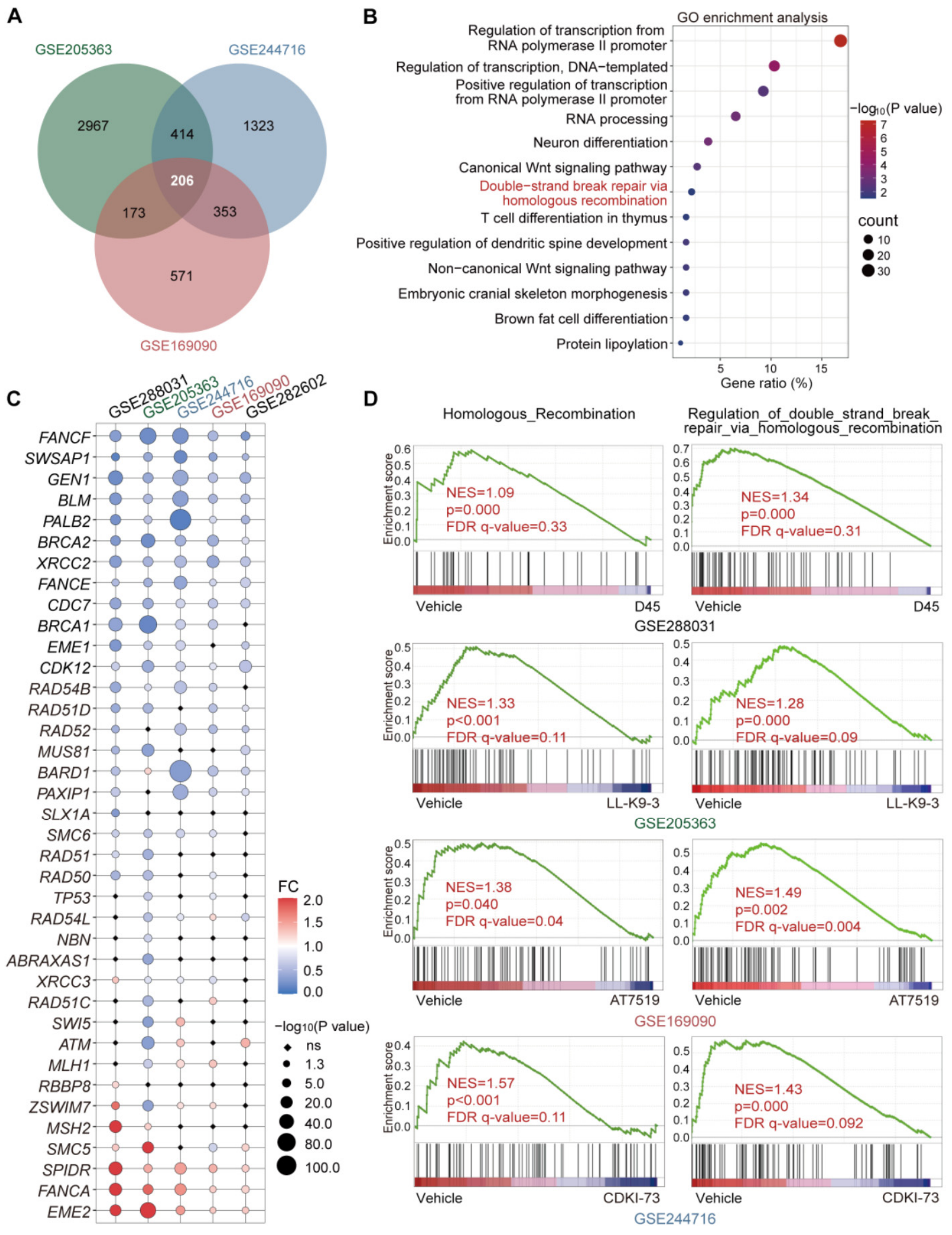

Supplementary Figure S2. Targeting CDK9 impaired the homologous recombination repair. A, Venn diagram of DEGs ($P < 0.05$, |Fold Change| ≥ 1.5) in PCa cells treated with CDK9 degrader or CDK9 inhibitors from GSE205363, GSE169090 and GSE244716 datasets. **B,** GO enrichment analysis on the 206 overlapping genes obtained from (**A**). **C,** Bubble plot displaying the expression patterns of HRR-related genes in PCa cells or breast cancer cells treated with CDK9 degraders or CDK9 inhibitors across multiple GEO datasets. **D,** GSEA of “homologous recombination” and “regulation of double strand break repair via homologous

recombination" gene sets in PCa cells treated with CDK9 degraders or CDK inhibitors from GSE288031 (this study using degrader D45), GSE205363 (using degrader LL-K9-3), GSE169090 (using inhibitor AT7519), and GSE244716 (using inhibitor CDKI-73). *P* values were determined by Hypergeometric test in **B**, Moderated *t*-test in **C**, and Permutation test in **D**.

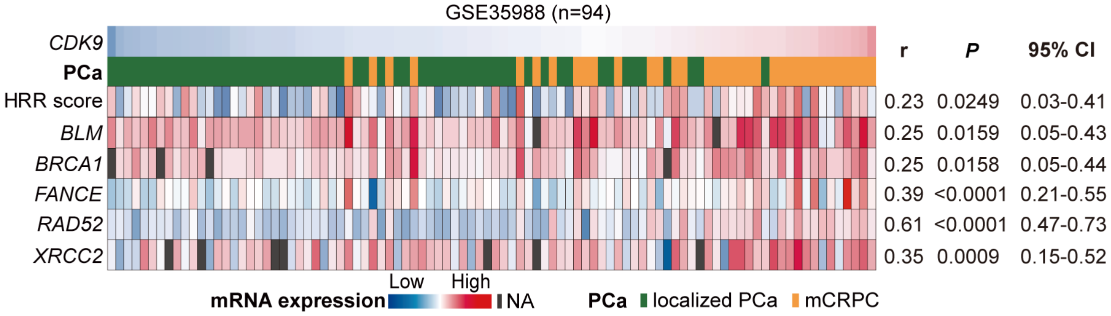

Supplementary Figure S3. *CDK9* expression was positively correlated with HRR activation. Heatmap showing the expressional correlation between *CDK9* and HRR genes in 94 human PCa specimens from GSE35988 dataset. The top bar represented the *CDK9* mRNA levels (from low to high), and the second bar indicated the corresponding pathological information as localized PCa (green) or metastatic CRPC (orange). The WP_Score for HRR signaling represented the HRR pathway activity by GSVA. mRNA levels of *BLM*, *BRCA1*, *FANCE*, *RAD52*, and *XRCC2* were also included. Pearson correlation coefficients (r) and *P* values were indicated for the associations between *CDK9* mRNA and other features. *P* values were determined by Pearson correlation coefficient test.

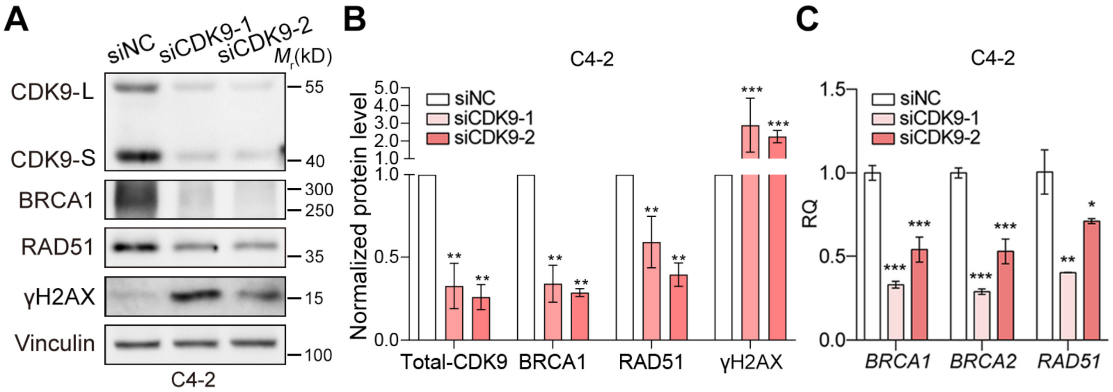

Supplementary Figure S4. CDK9 depletion by siRNAs downregulated HRR genes. A-B, Representative images (**A**) and quantification (**B**) for Western blots of CDK9 and HRR-related proteins in C4-2 cells transfected with siRNAs targeting two independent sequences of human CDK9 mRNA (siCDK9-1 and siCDK9-2) (n=3, mean ± SEM). siNC, siRNA control. Vinculin was used as the normalization control. **C,** qRT-PCR analyses of *BRCA1*, *BRCA2* and *RAD51* mRNA levels in C4-2 cells transfected with siRNAs targeting CDK9 for 48 h (mean ± SD). *P* values were determined by one-way ANOVA test in **B, C**. $*P < 0.05$, $**P < 0.01$, $***P < 0.001$.

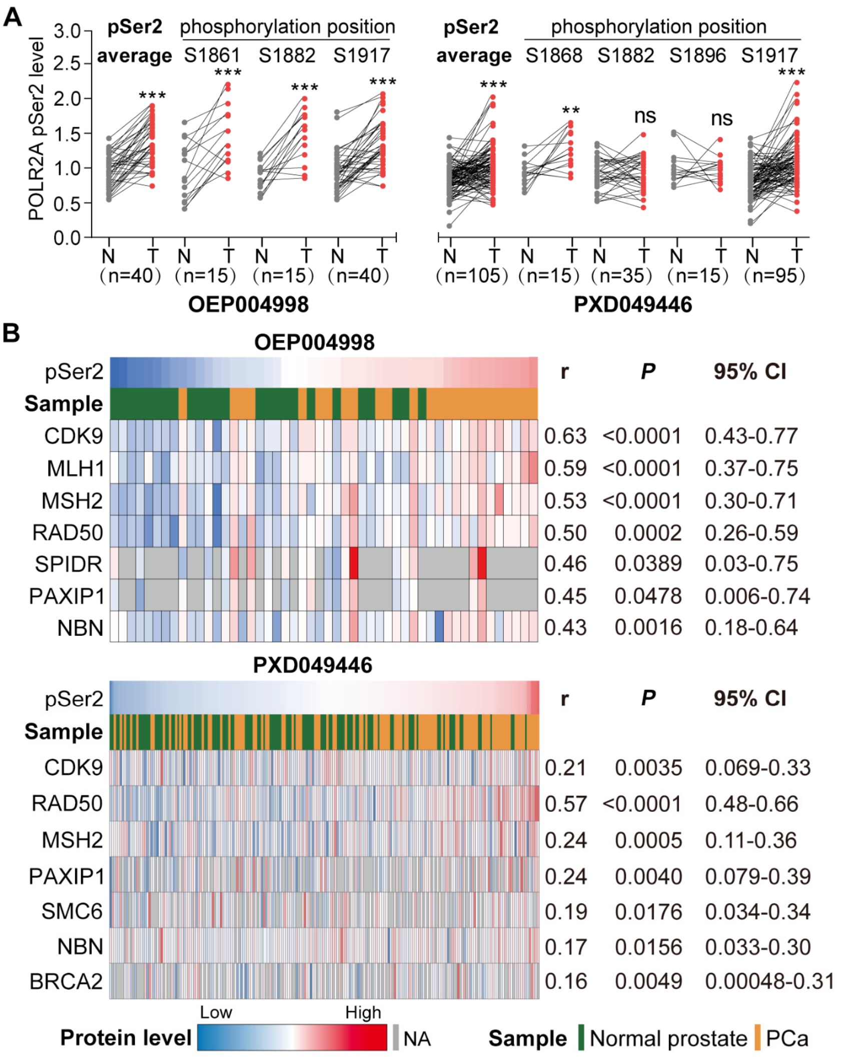

Supplementary Figure S5. Phosphorylation level at the Ser2 position (pSer2) of POLR2A (RNA Polymerase II Subunit A) was positively correlated with HRR activation in human PCa. A, pSer2 levels of POLR2A in public PCa proteomic datasets. Quantitative analysis of pSer2 levels was performed using OEP004998 dataset (n = 40 paired PCas; left panel) and PXD049446 dataset (n = 105 paired PCas; right panel). Since that phosphorylation at the Ser2 position was distributed across multiple tandem consensus heptapeptide repeats within the POLR2A C-terminal domain, the mean pSer2 levels were calculated for PCa samples and their corresponding normal prostate tissues. Specifically, the average pSer2 level was determined based on phosphorylation at S1861, S1882, and S1917 for OEP004998, and S1868, S1882, S1896, and S1917 for PXD049446. Data, mean ± SD; *P*, two-tailed paired Student's *t*-test. **B,** Correlation between pSer2 levels of POLR2A and HRR proteins in human PCa. Heatmap illustrating Pearson correlation coefficients (r) between the mean pSer2 level of POLR2A and HRR proteins in OEP004998 (n = 25 paired PCas) and PXD049446 (n = 105 paired PCas) datasets, in which CDK9 protein expression was detected. The top bar displayed the mean pSer2 levels of POLR2A (from low to high), while the second bar indicated pathological status as normal (green) or PCa (orange).

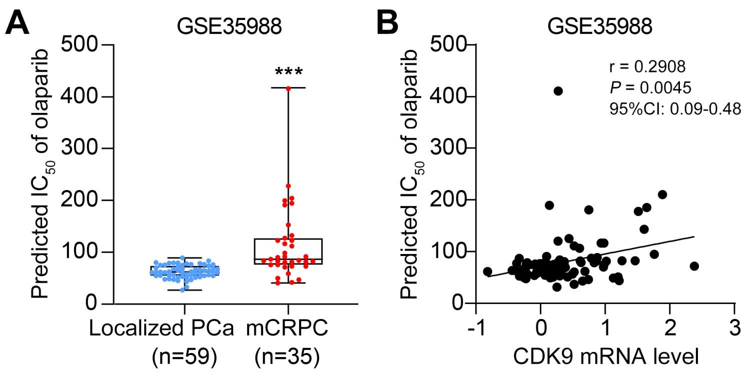

Supplementary Figure S6. High *CDK9* mRNA expression was associated with olaparib resistance in mCRPC. **A,** The predicted IC_{50} values of olaparib in human localized PCa (n = 59) and mCRPC (n = 35) samples from GSE35988 dataset. Data were represented as mean ± SD. *P* values were determined by Student's *t*-test. ****P* < 0.001. **B,** The correlation between *CDK9* mRNA levels and the predicted IC_{50} values of olaparib in GSE35988 dataset by Pearson correlation analysis.

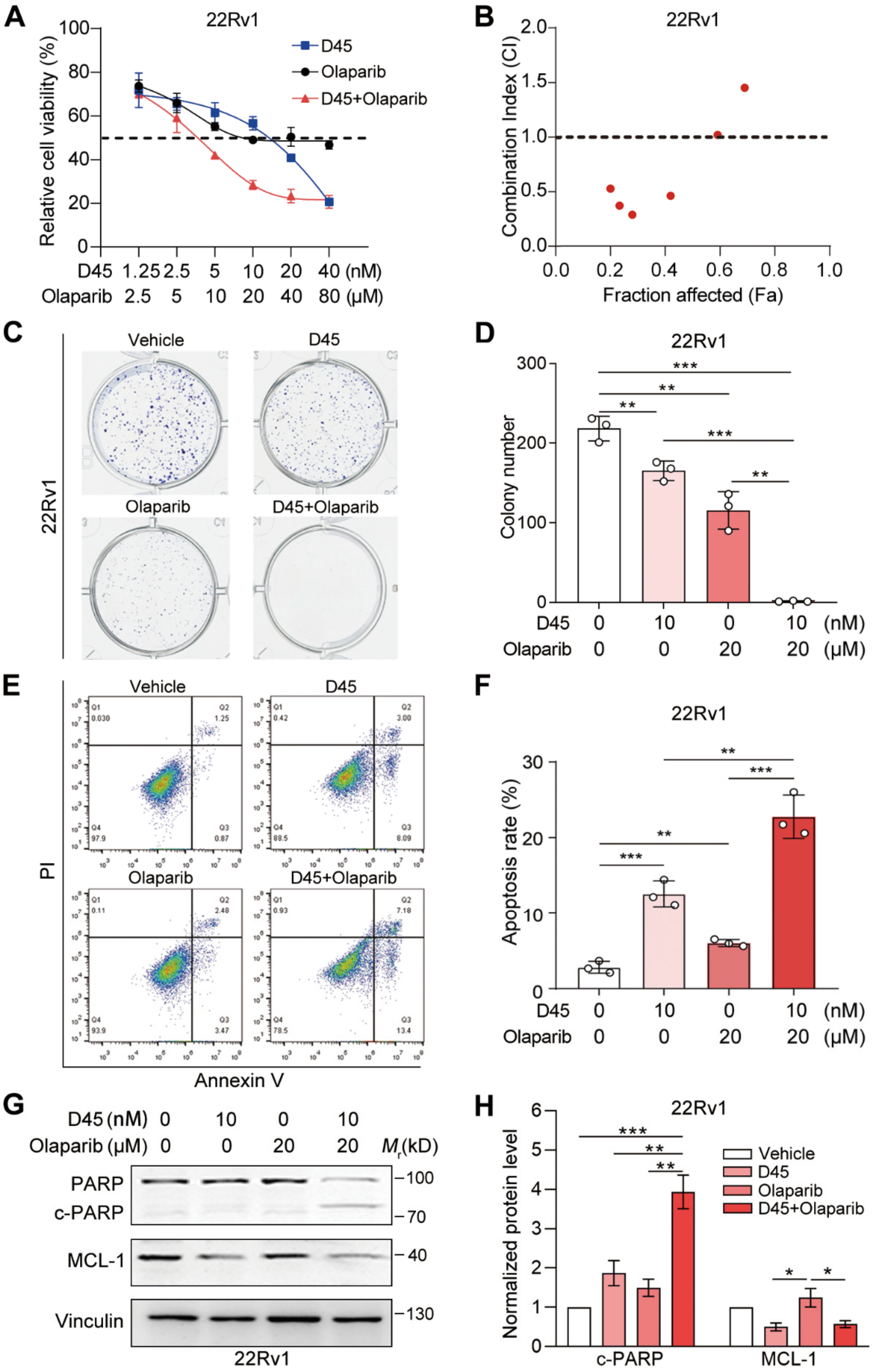

Supplementary Figure S7. D45 synergized with olaparib to decrease CRPC cell viability in 22Rv1 cells. A, Cell viability of 22Rv1 cells treated with D45 and olaparib, alone or combined, at the indicated concentrations for 72 h by MTT assay. **B,** Combination index for D45 and olaparib in 22Rv1 cells by Chou-Talalay analysis. **C,** Colony formation capability of 22Rv1 cells treated with D45 and olaparib, alone or combined, at the indicated

concentrations. **D,** Quantification of colony numbers in **C**. **E,** Apoptosis in 22Rv1 cells treated with D45 and olaparib, alone or combined, at the indicated concentrations for 72 h with the Annexin V/PI staining by flow cytometry. **F,** Quantification for the apoptosis percentage in **E**. **G-H,** Representative images (**G**) and quantification (**H**) for Western blots of cleaved PARP (c-PARP) and MCL-1 protein in 22Rv1 cells treated with D45 and olaparib, alone or combined, at the indicated concentrations for 72 h. Vinculin was used as the normalization control (n=3). Data were represented as mean ± SD in **D, F**, or mean ± SEM in **H**. *P* values were determined by two-way ANOVA test in **D, F,** and by one-way ANOVA test in **H**. $^{*}P < 0.05$, $^{**}P < 0.01$, $^{***}P < 0.001$.

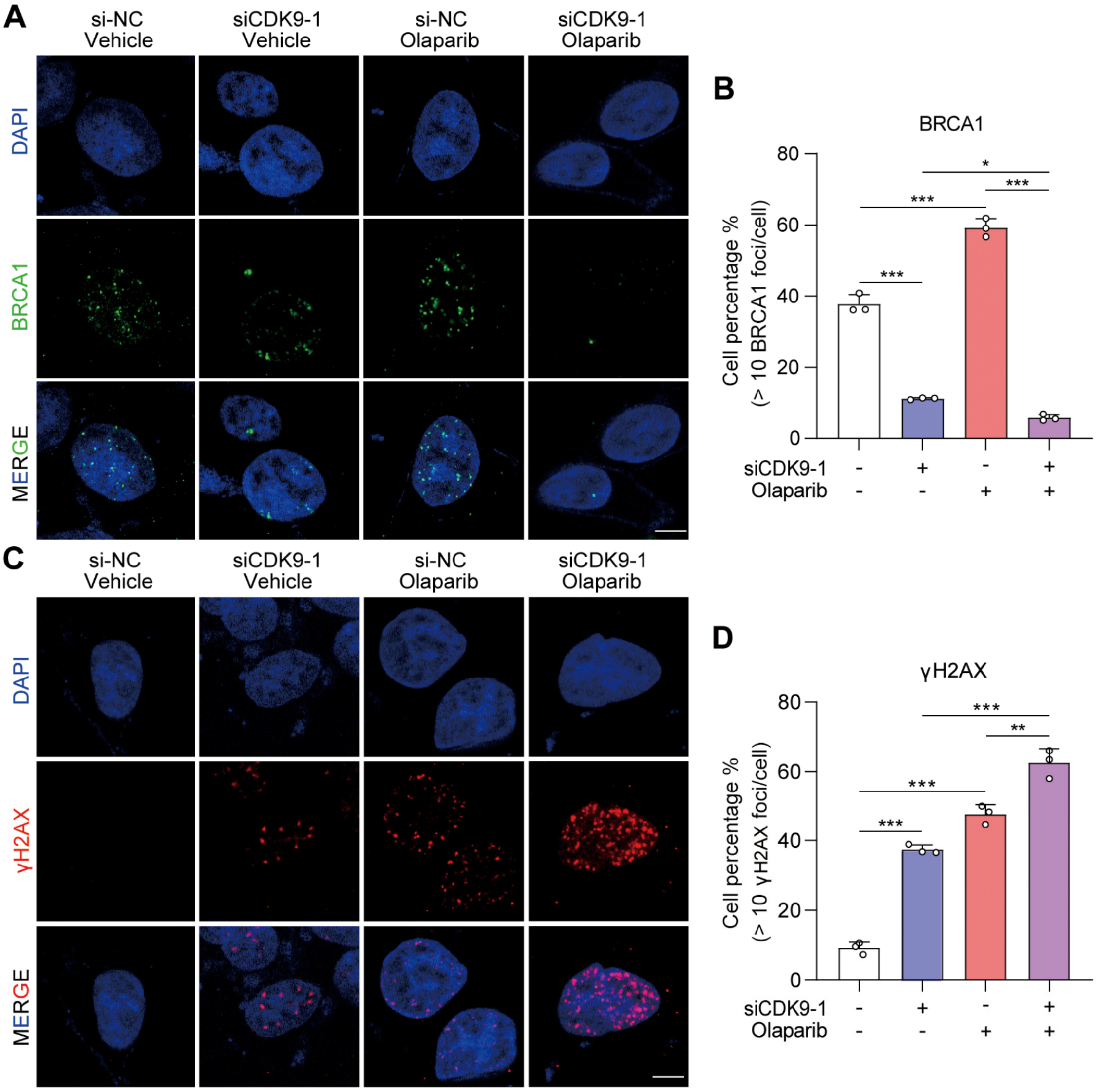

Supplementary Figure S8. CDK9 depletion by siRNA synergized with olaparib to enhance DNA damage. A, C, Representative images of BRCA1 (**A**) and γH2AX foci (**C**) in C4-2 cells treated with siCDK9-1 and olaparib, alone or combined, by immunofluorescence staining. DAPI was used for nuclei staining. Scale bar, 10 μm. **B, D,** The percentage of cells that had more than 10 BRCA1 foci per cell in (**A**) and more than 10 γH2AX foci per cell in (**C**). Data were represented as mean ± SD with P values determined by two-way ANOVA test in **B**, **D**. $^{*}P < 0.05$, $^{**}P < 0.01$, $^{***}P < 0.001$.

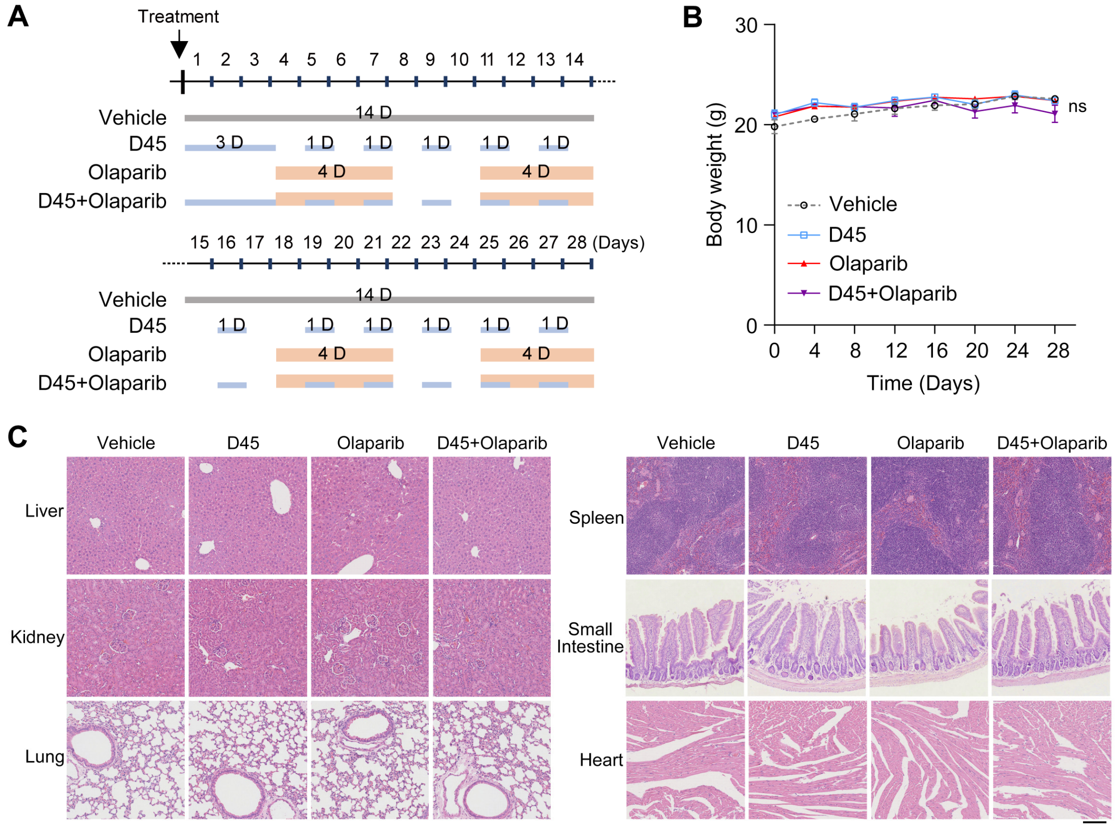

Supplementary Figure S9. A 28-day repeated-dose toxicity assessment on sequential administration of D45 and olaparib. A, Experimental scheme of sequential regimen for D45 and olaparib, alone or combined, in C57BL/6 mice for 28 days. The mice were divided into four groups: Vehicle, D45 (1.5 mg/kg), Olaparib (50 mg/kg), and D45+Olaparib (1.5 mg/kg and 50 mg/kg, respectively), n =3 per group. **B,** Body weight curve of mice with D45 and olaparib, alone or combined. **C,** The representative H&E images in multiple tissues from mice treated with D45 and olaparib, alone or combined. Scale bar, 100 μm. Data were represented as mean ± SD and *P* values were determined by two-way ANOVA test in **B**. ns, $P \geq 0.05$.

Supplementary Table S1. List of primers for qRT-PCR and CUT&Tag, and siRNAs

**Primers**		
Primers for qRT-PCR		
**Gene**	**Forward (5'-3')**	**Reverse (5'-3')**
ACTB	GTGTTGCCCCTGAAGAGCAT	GCTGGGACATTGAAAGTCTCA
BARD1	CTTTCATCCGAAGGCGGGA	TCAGAATGTTAGTACAACGCGA
BLM	TCTGCGTGCGAGGATTATGG	AGTCCTTGACCCTTTGCTGA
BRCA1	TGCTCTGGGTAAAGTTCATTGG	ACACTGTGAAGGCCCTTTCT
BRCA2	CCCAGCTTACCTTGAGGGTT	TCTGATGATGGACGCCAAAT
CDC7	CGTACTCCCTTAAACCCTGCTT	GCCATTGGCTCATCCATCTG
CDK1	AAACTACAGGTCAAGTGGTAGCC	TCCTGCATAAGCACATCCTGA
CDK4	CTGGTGTTTGAGCATGTAGACC	GATCCTTGATCGTTTCGGCTG
CDK9	ATAACCGCTGCAAGGGTAGTA	TTGACCAAAACATTGCTCAACAG
CDK10	CGGAGGCTCAGGTCAAGTG	CACACAACCCTTGTCGGTCA
CDK12	GGAGTCCACTCCCCAGTAGG	TGGAATGACGACTGCGTGAA
CDK16	TGACATTATCCACACGGAGAAGT	ACAGTTTCACGTTGTGCATGTT
FANCE	GTCCCAGCCAGATGGACTT	AGGTCAGGGCAGTTGTAAGC
FANCF	TGGAGACCTGTAAAGCGCAG	GCGCTGAGACCCAAAACTTG
MCL1	TGCTTCGGAAACTGGACATCA	TAGCCACAAAGGCACCAAAAG
MYC	CACCAGCAGCGACTCTGA	GATCCAGACTCTGACCTTTTGC
MYCL	CTGCGGGGAGGATTTCTACC	CATGCAGTCACGGCGTATGAT
PALB2	TGTGAGGAGAAGGAAAAGTTAAAGG	GGACTCAGGCCCAACATCAA
RAD51	AGACCGAGCCCTAAGGAGAG	GCATTGCCATTACTCGGTCC
RAD52	CCAGAAGGTGTGCTACATTGAG	ACAGACTCCCACGTAGAACTTG
XRCC2	TGCTTTATCACCTAACAGCACG	TGCTCAAGAATTGTAACTAGCCG
Primers for CUT&Tag		
**Gene**	**Forward (5'-3')**	**Reverse (5'-3')**
BRCA1	TCTCTGTCTCCAGCAATTG	GTATCAGGTAGGTGTCCAG
BRCA2	GAAGGAGATTAGACTCTGAGC	CCAAAATCTACAAACGTGGA
RAD51	GGAAACCAGAATCTGCAA	CTGCAGCACTTAAGGTTT
**siRNAs**		
**Name**	**Sense (5'-3')**	**Antisense (5'-3')**
siCDK1-1	GTATAAGGGTAGACACAAATT	TTTGTGTCTACCCTTATACTT
siCDK1-2	GGCACTGAATCATCCATATTTTT	AAATATGGATGATTCAGTGCCTT
siCDK4-1	CTCTTATCTACATAAGGATTT	ATCCTTATGTAGATAAGAGTT
siCDK4-2	AAGGTAATCCGGAGTGAGCAATT	TTGCTCACTCCGGATTACCTTTT
siCDK9-1	GGCCAAACGTGGACAACTATT	TAGTTGTCCACGTTTGGCCTT
siCDK9-2	GUGAUGAAGUUUCCAAAUATT	UAUUUGGAAACUUCAUCACTT
siCDK10-1	CGAACATCGTGGAGCTGAATT	TTCAGCTCCACGATGTTCGTT
siCDK10-2	CCAGCCTCCTGGAGAATATTT	ATATTCTCCAGGAGGCTGGTT
siCDK16-1	TGAGATTGGCTTTGGGAAATT	TTTCCCAAAGCCAATCTCATT
siCDK16-2	AAGGAGATCAGACTGGAACATTT	ATGTTCCAGTCTGATCTCCTTTT
siNC	UUCUCCGAACGUGUCACGUTT	ACGUGACACGUUCGGAGAATT

Supplementary Table S2. List of antibodies

Antibodies	Company	Cat #	Dilution	RRID
β-Actin	Sigma-Aldrich	A1978	1:2,000 (WB)	AB_476692
BRCA1	Proteintech	22362-1-AP	1:1,000 (WB); 1:200 (IF/IHC)	AB_2879090
BRCA2	BOSTER	BA0668	1:1,000 (WB)	NA
Cleaved Caspase-3 (Asp175)	CST	9661	1:1,000 (WB) 1:200 (IHC)	AB_2341188
CDK9	CST	2316	1:200 (IHC)	AB_2291505
	ABclonal	A0886	1:1,000 (WB)	AB_2757443
γH2AX	ABclonal	AP0687	1:1,000 (WB); 1:200 (IF/IHC)	AB_2863808
Ki-67	Abcam	ab15580	1:400 (IHC)	AB_443209
MCL-1	Santa Cruz	sc-69839	1:1,000 (WB)	AB_1126067
c-Myc	Santa Cruz	sc-40	1:1,000 (WB)	AB_627268
RAD51	ABclonal	A6268	1:1,000 (WB); 1:200 (IHC)	AB_2766874
p-RNA Pol II CTD (Ser2)	Thermo	703108	1:100 (CUT&Tag)	AB_2784561
p-RNA POLR2A-S2	ABclonal	AP0749	1:1,000 (WB)	AB_2771441
RNA POLR2A	ABclonal	A2107	1:1,000 (WB)	AB_2764126
PARP	CST	9542	1:1,000 (WB)	AB_2160739
Vinculin	Proteintech	66305-1-Ig	1:1,000 (WB)	AB_2810300
Alexa 488-conjugated 2nd Ab	ABclonal	AS053	1:1,000 (IF)	AB_2768320
Alexa 555-conjugated 2nd Ab	ABclonal	AS057	1:1,000 (IF)	AB_2768321

Notes:

CST, Cell Signaling Technologies; IF, Immunofluorescence; IHC, Immunohistochemistry; Thermo, ThermoFisher Scientific; WB, Western blotting.

Supplementary Table S3. List of chemicals and kits

Items	Company	Cat #	Concentration
BCA protein assay kit	Thermo	23225	NA
BD apoptosis detection kit	BD Biosciences	556547	NA
Bovine serum albumin (BSA), fraction V	Sangon Biotech	A600332	5% in TBST
ChamQ universal SYBR qPCR master mix	Vazyme	Q311-02	NA
Crystal violet	Sigma-Aldrich	C0775	0.5%
DAB kit	MXB Biotechnologies	DAB-2031	NA
DAPI	Beyotime	P0131	5 μg/mL
DMSO	Sigma-Aldrich	D2650	NA
Enhanced ECL chemiluminescent substrate	Tanon Science & Technology	180-5001	NA
Fetal bovine serum, FBS	Gibco	1619646	10%
Formaldehyde solution	Beyotime	P0099	4%
Goat serum	Solarbio	SL038	5%
Hifair advance fast 1st strand cDNA synthesis superMix for qPCR	YEASEN	11149ES60	NA
Lipofectamine RNAiMAX	Thermo	13778150	2 μL/mL
MTT	Sigma-Aldrich	M2128	0.5 mg/mL
Nitrocellulose membrane	Merck	GE10600002	NA
NovoNGS CUT&Tag 4.0 high-sensitivity kit	Novoprotein	N259-YH01	NA
Olaparib	MedChemExpress	HY-10162	1.5 mg/kg (*in vivo*); 20-80 μM (*in vitro*)
Opti-MEM	Thermo	31985070	NA
Protease inhibitor cocktail	Abmole	M5293	NA
Phosphatase inhibitor cocktail	Abmole	M7528	NA
RIPA	Beyotime	P0013C	NA
Trizol	Thermo	15596018CN	NA
UltraSensitive SP (Rabbit) IHC kit	MXB Biotechnologies	KIT-9707	NA

Supplementary Table S4. Hematological and serum biochemical profiles of C57BL/6 mice with repeated-dose sequential treatment by D45, olaparib, or their combination

Classification	Parameter	Vehicle	D45	Olaparib	D45+Olaparib
Leukocyte System	WBC (10^9/L)	6.40 ± 0.30	2.97± 0.56***	3.20 ± 1.12***	1.58 ± 0.75***
	NEUT (10^9/L)	0.56 ± 0.07	0.40 ± 0.08	0.42 ± 0.16	0.32 ± 0.12*
	LYMPH (10^9/L)	5.54 ± 0.20	2.36 ± 0.59***	2.66 ± 0.94***	1.18 ± 0.62***
	MONO (10^9/L)	0.15 ± 0.04	0.12 ± 0.03	0.06 ± 0.04*	0.04 ± 0.04**
	EO (10^9/L)	0.14 ± 0.02	0.08 ± 0.03	0.06 ± 0.03*	0.04 ± 0.05**
	BASO (10^9/L)	0.00 ± 0.00	0.00 ± 0.00	0.00 ± 0.00	0.00 ± 0.00
Erythroid System	RBC (10^{12}/L)	9.69 ± 0.59	10.41 ± 0.41	10.49 ± 0.15	11.30 ± 1.04*
	HGB (g/L)	140.33 ± 7.64	149.00 ± 6.56	150.33 ± 2.52	159.80 ± 14.50
	HCT (%)	45.27 ± 2.27	48.20 ± 1.30	48.00 ± 0.89	50.72 ± 4.73
	MCV (fL)	46.70 ± 0.66	46.33 ± 0.68	45.73 ± 0.29	44.88 ± 0.51**
	MCH (pg)	14.50 ± 0.35	14.33 ± 0.12	14.30 ± 0.10	14.16 ± 0.25
	MCHC (g/L)	310.00 ± 8.54	309.33 ± 6.43	313.00 ± 1.00	315.20 ± 4.44
	RDW-SD (fL)	28.17 ± 1.78	25.60 ± 0.72	25.03 ± 0.65	24.38 ± 2.23*
	RDW-CV (%)	19.13 ± 0.51	18.90 ± 0.52	18.73 ± 0.29	19.62 ± 0.81
	RET (%)	3.95 ± 0.83	3.17 ± 0.24	3.64 ± 0.27	4.28 ± 1.37
	RET (10^9/L)	379.83 ± 61.63	329.00 ± 15.62	381.37 ± 22.71	486.92 ± 179.63
Thrombocyte System	PLT (10^9/L)	1096.00 ± 325.72	1544.00 ± 71.19	1606.00 ± 40.93	1729.00 ± 453.36
	MPV (fL)	6.63 ± 0.35	6.40 ± 0.00	6.30 ± 0.00	6.44 ± 0.18
	PDW (fL)	6.53 ± 0.40	6.53 ± 0.06	6.40 ± 0.10	6.68 ± 0.24
Serum Biochemical Marker	ALT (U/L)	38.30 ± 13.94	32.10 ± 2.19	27.93 ± 3.15	48.88 ± 11.22
	AST (U/L)	114.50 ± 54.81	95.50 ± 16.10	89.93 ± 19.06	129.20 ± 35.49
	CREA (µmol/L)	8.33 ± 4.04	4.00 ± 1.00	6.67 ± 1.53	4.00 ± 0.82
	UREA (mmol/L)	7.77 ± 1.59	7.50 ± 0.44	7.57 ± 0.25	7.08 ± 1.47

Notes:

1. *P* values: drug-treated group *vs.* vehicle group by one-way ANOVA test (n = 3 per group). *, $P < 0.05$; **, $P < 0.01$; ***, $P < 0.001$.
2. WBC, white blood cell; NEUT, neutrophil; LYMPH, lymphocyte; MONO, monocyte; EO, eosinophil; BASO, basophil; RBC, red blood cell; HGB, hemoglobin; HCT, hematocrit; MCV, mean corpuscular volume; MCHC, mean corpuscular hemoglobin concentration; RDW-SD, red cell distribution width - standard deviation; RDW-CV, red cell distribution width - coefficient of variation; RET, reticulocyte; PLT, platelet; MPV, mean platelet volume; PDW, platelet distribution width; ALT, alanine aminotransferase; AST, aspartate aminotransferase; CREA, creatinine; UREA, urea.
